# Impaired adenosine pathways in HFpEF: insights into cardiorenal alterations and endothelial responses

**DOI:** 10.3389/fphar.2026.1720123

**Published:** 2026-02-11

**Authors:** Maria Antonietta Riemma, Gennaro Madonna, Elena Mele, Elena Conte, Marialucia Telesca, Giacomo De Palma, Barbara Kutryb-Zając, Carla Cicala, Paola Imbrici, Antonella Liantonio, Antonella De Angelis, Konrad Urbanek, Liberato Berrino, Armando Ialenti, Donato Cappetta, Maria Donniacuo, Elisabetta Caiazzo

**Affiliations:** 1 Department of Experimental Medicine, University of Campania “Luigi Vanvitelli”, Naples, Italy; 2 Department of Pharmacy, School of Medicine and Surgery, University of Naples “Federico II”, Naples, Italy; 3 Department of Pharmacy-Drug Sciences, University of Bari “Aldo Moro”, Bari, Italy; 4 Department of Biochemistry, Medical University of Gdansk, Gdańsk, Poland; 5 Centre of Experimental Cardiooncology, Medical University of Gdansk, Gdańsk, Poland; 6 Department of Molecular Medicine and Medical Biotechnologies, University of Naples “Federico II”, CEINGE-Advanced Biotechnologies, Naples, Italy; 7 Department of Experimental Medicine, University of Salento, Lecce, Italy; 8 School of Infection & Immunity, College of Medical, Veterinary and Life Sciences, University of Glasgow, Glasgow, United Kingdom

**Keywords:** adenosine deaminase inhibition, adenosine metabolism, adenosine receptors, adenosine signaling, cardiorenal crosstalk, endothelial activation, heart failure with preserved ejection fraction, oxidative stress

## Abstract

**Introduction:**

Heart failure with preserved ejection fraction (HFpEF) accounts for nearly half of all heart failure cases and lacks effective therapies. Key features of HFpEF include endothelial dysfunction, fibrosis, and oxidative stress. Adenosine signaling, regulated by enzymes and receptors, is critical for vascular homeostasis and inflammation, but its role in HFpEF remains poorly understood. Adenosine receptors are abundantly expressed in the heart and kidney, modulating vascular, fibrotic, and tubular processes. Dysregulation of adenosine pathways in either organ may drive hypertension, microvascular dysfunction, and maladaptive cardio-renal crosstalk, highlighting the need to investigate adenosine signaling as a combined multi-organ target.

**Methods:**

HFpEF was induced in Dahl salt-sensitive rats by high-salt diet. Cardiac structure, function, fibrosis, oxidative stress, cytokines, renal adenosine receptors and cardiac adenosine pathway components were assessed using echocardiography, histology, proteome profiling and Western blotting. Human cardiac microvascular endothelial cells were treated with endothelin-1 in the presence of selective A_2A_ or A_2B_ agonists, or adenosine deaminase (ADA) inhibition, and profibrotic/oxidative stress genes were analyzed by qPCR.

**Results:**

Hypertensive rats exhibited diastolic dysfunction with preserved systolic function, cardiac and renal fibrosis, oxidative/nitrative stress, and elevated pro-inflammatory cytokines. Cardiac expression of CD39, CD73, and ADA enzymes was significantly reduced, indicating impaired adenosine metabolism, while transporters ENT2 and CNT2 were also downregulated, reflecting impairment of both equilibrative and concentrative adenosine transport. Adenosine receptor profiles were altered: A_1_ expression increased, A_2A_ decreased, and A_2B_ and A_3_ selectively upregulated in hypertensive, but not HFpEF, animals. In the kidney, A_1_ and A_2A_ receptor expression showed region-specific, time-dependent changes. In human endothelial cells, A_2A_ activation or ADA inhibition suppressed endothelin-1-induced COL1, COL3, and TGF-β1 expression, whereas A_2B_ had no effect. Both A_2A_ and A_2B_ restored MnSOD expression, while NOX4 was selectively increased by A_2B_. Only A_2A_ activation induced CAT expression, highlighting its stronger antioxidant role.

**Conclusion:**

Impaired adenosine metabolism and transport, along with altered receptor signaling contribute to HFpEF progression. Selective A_2A_ activation attenuates endothelial pro-fibrotic profiles and restores antioxidant defenses, supporting its therapeutic potential. Renal receptor changes reinforce maladaptive cardio-renal crosstalk, emphasizing the importance of multi-organ adenosine modulation in HFpEF and encouraging further translational studies.

## 1 Introduction

Heart failure with preserved ejection fraction (HFpEF) accounts for approximately half of all HF cases and represents a major clinical challenge due to its increasing prevalence and the scarcity of therapeutic options (Borlaug, 2020). Unlike HF with reduced EF (HFrEF), HFpEF is characterized by normal or near-normal left ventricular (LV) EF accompanied by diastolic dysfunction, endothelial impairment, and myocardial fibrosis (Paulus and Tschope, 2013; Shah et al., 2020). Studies have shown that 70% of patients with HF subsequently develop the disease in other vital organs including the kidneys. Several factors contribute to the progression of cardiac and renal damage, such as inadequate perfusion, inflammation, neurohumoral imbalance, and oxidative stress (Vallon et al., 2008; Sharma et al., 2025). Despite significant advances in understanding its multifactorial pathophysiology, therapeutic options remain limited to sodium-glucose co-transporter 2 inhibitors (SGLT2i) and symptom management (Dunlay et al., 2017; McDonagh et al., 2023).

Adenosine, an endogenous purine nucleoside released in response to metabolic stress, inflammation, hypoxia, and tissue injury, has emerged as a molecular player involved in HFpEF (Eltzschig et al., 2012). Extracellular adenosine is mainly generated via enzymatic degradation of ATP by CD39 and CD73, and its signaling is tightly regulated by the interaction with four G-protein-coupled receptors: A_1_, A_2A_, A_2B_, and A_3_ (Hasko et al., 2008). In addition to enzymatic production and receptor signaling, extracellular adenosine availability is dynamically controlled by nucleoside transporters, including equilibrative (ENTs) and concentrative (CNTs) transporters, which regulate adenosine flux across the plasma membrane and thereby shape the magnitude and duration of receptor activation (Gray et al., 2004; Pastor-Anglada and Pérez-Torras, 2018a; Pastor-Anglada and Pérez-Torras, 2018b). Adenosine receptors regulate coronary blood flow, vascular permeability, inflammation, and fibrosis (Fredholm et al., 2011; Caiazzo et al., 2016; Ialenti et al., 2018; Antonioli et al., 2023). In particular, the A_2A_ and A_2B_ receptors are abundantly expressed on endothelial cells, where they play key roles in modulating vascular tone, reducing pro-inflammatory cytokine release, and maintaining endothelial barrier integrity (Narravula et al., 2000; Haskó et al., 2008). A_2A_ receptor activation has been shown to inhibit leukocyte adhesion and cytokine production, while A_2B_ receptor activation, typically upregulated during hypoxia or inflammation, promotes nitric oxide (NO) release and stabilizes the endothelial barrier (Olanrewaju and Mustafa, 2000; Linden and Cekic, 2012; Ralevic and Dunn, 2015). Adenosine also plays a critical role in the kidney by regulating renal blood flow, glomerular filtration rate (GFR), tubuloglomerular feedback, and renin secretion (Oyarzun et al., 2017). All four adenosine receptor subtypes are expressed in the kidney (Vallon et al., 2006; Vallon et al., 2008; Wissmann et al., 2025). Functionally, exogenous adenosine reduces the GFR by narrowing the afferent arterioles, particularly in the nephrons, thus decreasing the kidney’s salt burden and transport activity. Furthermore, adenosine promotes vasodilation in the cortex and medulla, modulates sodium chloride transport throughout the nephron and collecting ducts (Vallon and Osswald, 2009) and inhibits renin release (Ortiz-Capisano et al., 2013). Dysregulation of adenosine signaling is increasingly recognized in cardiovascular diseases, including hypertension, atherosclerosis, and HF, therefore alterations in adenosine metabolism or receptor expression may contribute to cardiovascular disease progression (Asakura et al., 2007; Khodadadi et al., 2014; He et al., 2014; Antonioli et al., 2023; Qian et al., 2025). Preclinical studies suggest that adenosine receptor subtypes exert a range of effects relevant to HFpEF pathophysiology. For instance, A_1_AR activation may reverse cardiac hypertrophy and remodeling, improve mitochondrial function, and enhance calcium handling (Mubagwa and Flameng, 2001; Kilpatrick et al., 2002), A_2A_AR appears to attenuate cardiac inflammation, fibrosis, and hypertrophy while promoting vasodilation (Yang et al., 2006). Despite these promising preclinical findings, clinical translation has so far been disappointing. Notably, neladenoson, a partial A_1_ receptor agonist, failed to demonstrate significant improvement in exercise capacity in HFpEF patients (Shah et al., 2019). Such discrepancies may be explained by disease heterogeneity, comorbidities, and pharmacological interactions. These observations highlight the gap between experimental evidence and clinical efficacy, underscoring the need for a better understanding of adenosine signaling in HFpEF.

Renal and cardiovascular diseases share common risk factors and are interconnected through the cardiorenal axis, a pathophysiological and clinical relationship in which dysfunction in one organ can lead to dysfunction in the other. Collectively, the cardiovascular and renal roles of adenosine highlight its dual contribution to vascular homeostasis and kidney function and suggest that dysregulated adenosine metabolism or receptor signalling may drive maladaptive cardio-renal crosstalk in HFpEF.

On this premise, the objective of the present study was to investigate changes in the expression of enzymes and receptors involved in adenosine signaling in an *in vivo* model of HFpEF using Dahl salt-sensitive rats, a well-established model that recapitulates the hypertensive and fibrotic multi-organ phenotype of human HFpEF (Cappetta et al., 2019; Urbanek et al., 2023; Conte et al., 2025), focusing on heart and kidney. Additionally, we evaluated the effects of selective activation of A_2A_ and A_2B_ adenosine receptors and the inhibition of ADA on cardiac endothelial cells *in vitro*, in the presence of endothelin-1, used to simulate an inflammatory and vasoconstrictive environment. By combining *in vivo* and *in vitro* approaches, our study aims to provide new insights into the role of adenosine signaling in HFpEF and its potential as a therapeutic target.

## 2 Materials and methods

### 2.1 Animal model of heart failure with preserved ejection fraction (HFpEF)

Male Dahl salt-sensitive rats (7 weeks old, n = 6/group) obtained from Charles River Laboratories (Wilmington, MA, United States), were maintained under a strict 12-h light/dark cycle within an environment with controlled temperature and humidity. Animals were fed a high-salt diet (8% NaCl) for 5 weeks to induce hypertension (12HS group) and then continued for the following 5 weeks to develop HFpEF (17HS group). The control group received a diet with a 0.3% NaCl (low-salt diet, LS group). All procedures related to animal housing and treatment were carried out in accordance with the national ethical guidelines and the European Directive 2010/63/EU. The study protocol was approved by the Italian Ministry of Health (authorization no. 582/2015-PR) and by the local ethics committee.

### 2.2 Blood pressure and heart function

Average arterial pressure was monitored on a weekly basis in conscious animals utilizing the non-invasive tail-cuff method. Echocardiographic evaluations were carried out using a high-resolution Micro-Ultrasound System (Vevo 3100 VisualSonics Inc., Ontario, Canada). Rats were anesthetized with inhaled isoflurane, induced at 3% and subsequently maintained at 1.5%–2.5% range during experimental procedure and the animal’s body temperature was maintained at 37 °C with the aid of a warming surface. Serial M-mode recordings were acquired along the minor axis at the papillary muscle level to assess LV diastolic diameter and wall thickness, which were then employed to calculate EF and fractional shortening (FS). To ensure objectivity, a lone researcher, unaware of the animal groupings, conducted all image acquisitions and subsequent offline analyses. Before the animals were euthanized, hemodynamic variables were measured. In anesthetized subjects, the right carotid artery was cannulated with a microtip pressure sensor (SPR-612, Millar Instruments, Houston, TX, United States) linked to an A/D converter (iWorx 214) and a computer interface. The catheter was advanced into the left ventricular cavity to assess LV pressures and the positive and negative first derivative of pressure over time (+dP/dt and -dP/dt) in the intact chest configuration. Animals were euthanized under deep isoflurane anesthesia (5%), in accordance with institutional guidelines and approved experimental protocols.

### 2.3 Cytokines and chemokines protein array

Cardiac homogenates prepared from left ventricular tissue as detailed below, were evaluated using the Proteome Profiler Rat XL Cytokine Array (ARY030, R&D Systems, Milan, Italy) to simultaneously detect multiple cytokines and chemokines. Equal volumes (1.5 mL) of homogenates from the different experimental groups were incubated with the pre-coated array membranes according to the manufacturer’s protocol. Signal detection was performed with an enhanced chemiluminescence (ECL) kit, visualized using the ChemiDoc Imaging System (Bio-Rad, Milan, Italy), and quantified with Image Lab software (Bio-Rad).

### 2.4 Histological analysis

Excised hearts and kidneys were fixed in 4% paraformaldehyde for 1 h, cryoprotected in 30% sucrose overnight at 4 °C, and embedded in optimal cutting temperature (OCT) compound. Serial sections (10 µm) of left ventricles and whole kidneys were prepared using a cryostat (Leica Microsystems, Wetzlar, Germany). Cardiac and renal tissue were stained with Masson’s trichrome to evaluate fibrosis. For cardiac tissue, to calculate the extent of perivascular fibrosis, the area of perivascular accumulation of collagen was measured and expressed as a ratio to the total area of the vascular wall. Interstitial fibrosis was expressed as the area fraction of sampled tissue occupied by the collagen deposits. Measurements were done using ImageJ software (Media Cybernetics, Rockville, MD, United States) following the staining with Masson’s trichrome. The quantification included 50 vascular profiles analyzed in each experimental group. Assessment of reactive oxygen species (ROS) production was conducted via dihydroethidium (DHE) staining (Sigma-Aldrich). Peroxynitrite formation was evaluated by immunostaining with an anti-3-nitrotyrosine primary antibody (Millipore), followed by a FITC-conjugated secondary antibody (Jackson ImmunoResearch). Fluorescence images were acquired using a Zeiss LSM700 confocal microscope (Zeiss, Oberkochen, Germany).

### 2.5 Western blotting

Protein analysis of the adenosine pathway and adenosine receptors was performed in the left ventricle of the heart, as well as in the renal cortex, outer medulla, and inner medulla of Dahl rats using Western blotting. In addition, cardiac expression of endothelin-1 and VCAM-1 was evaluated. The samples were homogenized in RIPA lysis buffer (ChemCruz, Dallas, United States) containing protease and phosphatase inhibitor cocktails. The resulting lysate was centrifuged at 10,000 × g for 15 min at 4 °C, and the supernatant was collected for protein determination using the Bio-Rad protein assay (Bio-Rad), with bovine serum albumin (BSA) as the standard. Protein samples (30–35 μg) were separated by SDS-polyacrylamide gel electrophoresis, and the proteins were transferred to nitrocellulose membranes. The membranes were blocked for non-specific sites by washing with 0.1% PBS-Tween containing 5% w/v nonfat dry milk for 1 h at room temperature. After blocking, the membranes were incubated with the relevant primary antibody overnight at 4 °C. The following primary antibodies were used: anti-Adenosine A_1_ receptor (Santa Cruz Biotechnology, AB_2133860; 1:1000), anti-Adenosine A_2A_ receptor (Santa Cruz Biotechnology, AB_10858872; 1:1000), and anti-vinculin (Santa Cruz Biotechnology, AB_1131294; 1:1000), which were applied to renal cortex, outer medulla, and inner medulla samples. For cardiac tissue analysis, the antibodies used were: anti-CD39 (Novus, NBP2-25223, 1:1000), anti-CD73 (Novus, NBP1-85740, 1:1000), anti-adenosine deaminase (ADA; Novus, NBP1-87404, 1:500), anti-ENT2 (Proteintech, 26082-1-AP; 1:500), anti-CNT2 (Invitrogen, PA5-101897; 1:500), anti-Adenosine A_1_ receptor (Novus, NB300-549, 1:1000), anti-Adenosine A_2A_ receptor (Novus, NBP1-39474, 1:1000), anti- Adenosine A_2B_ receptor (Novus, NBP2-41312, 1:2000), anti- Adenosine A_3_ receptor (Novus, NLS-689, 1:1000), anti-endothelin-1(Abcam, ab18981, 1:1000), anti-VCAM1 (Abcam, ab134047,1:1000) and GAPDH (Sigma, G8795-100UL, 1:10000). After incubation, the membranes were washed three times with 0.1% PBS-Tween and incubated for an hour and a half with specific secondary antibodies conjugated with horseradish peroxidase (anti-rabbit, HAF008, R&D Systems, diluted 1:1000 or anti-mouse, Jackson 115-035-003, diluted 1:10000). The protein bands were normalized to the intensity of GAPDH or Vinculin, and the immunoreactive bands were detected using the enhanced chemiluminescence detection kit (ECL) and the Chemidoc imaging system (Bio-Rad). Densitometry was performed using ImageLab software.

### 2.6 Adenosine levels

Adenosine levels in cardiac homogenates were quantified using a fluorometric Adenosine Assay Kit (Cell Biolabs, San Diego, CA, United States), according to the manufacturer’s instructions.

### 2.7 Cell culture

Primary human cardiac microvascular endothelial cells (HCMECs; PromoCell, Heidelberg, Germany) were cultured in Endothelial Cell Growth Medium MV (PromoCell) following the manufacturer’s instructions. Culture medium was replaced every 48 h, and cell confluence was monitored by light microscopy. Cells were passaged at 80%–90% confluence and used for experiments between passages three and 5.

### 2.8 ET-1 treatment

HCMECs were seeded in 6-well plates (Falcon, Corning, NY) at a density of 0.5 × 10^6^ cells per well. Cells were pre-incubated for 60 min with either the adenosine A_2A_ receptor agonist, (3-(4-(2-((6-amino-9-((2R,3R,4S,5S)-5-(ethylcarbamoyl)-3,4-dihydroxytetrahydrofuran-2-yl)-9H-purin-2-yl)amino)ethyl)phenyl)propanoic acid hydrochloride; CGS21680, 10 µM), the adenosine A_2B_ receptor agonist (2-((6-amino-3,5-dicyano-4-(4-(cyclopropylmethoxy)phenyl)-2-pyridinyl)thio)acetamide; BAY60-6583, 10 µM), or the adenosine deaminase inhibitor (erythro-9-(2-hydroxy-3-nonyl)adenine hydrochloride, EHNA, 10 µM), prior to stimulation with endothelin-1 (ET-1, 100 nM; Origene, Rockville, United States). Cells treated with 0.05% dimethyl sulfoxide (DMSO) served as vehicle controls. After 6 h of stimulation, cells were harvested for RNA isolation and quantitative real-time PCR (qPCR) analysis.

### 2.9 RNA isolation and qPCR

Total RNA from HCMEC and kidney tissue fractions, including the cortex, outer medulla, and inner medulla, was isolated using TRIzol reagent according to the manufacturer’s recommendations (Bio-Rad). One microgram of RNA was reverse-transcribed into cDNA using the iScript Reverse Transcription Supermix for RT-qPCR (Bio-Rad). Samples were loaded in triplicate on a CFX96 Real-Time PCR Detection System (Bio-Rad) with SYBR Green Master Mix (Bio-Rad). The relative gene expression was determined using the 2^-ΔΔCt^ method. All data were compared to the respective controls (LS or unstimulated cells) and normalized to the glyceraldehyde-3-phosphate dehydrogenase (Gapdh) housekeeping gene for HCMEC and to 2-beta-microglobulin (2βm) gene for renal fractions. The following primers were used:

Collagen1 (*COL-1*): forward 5′-GAT​TCC​CTG​GAC​CTA​AAG​GTG​C-3′; reverse 5′- AGC​CTC​TCC​ATC​TTT​GCC​AGC​A -3′
Collagen 3 (*COL-3*): forward 5′-TGG​TCT​GCA​AGG​AAT​GCC​TGG​A-3′; reverse 5′-TCT​TTC​CCT​GGG​ACA​CCA​TCA​G-3′
Transforming Growth Factor Beta 1 (*TGF-β1*): forward 5′-TAC​CTG​AAC​CCG​TGT​TGC​TCT​C-3′; reverse 5′- GTT​GCT​GAG​GTA​TCG​CCA​GGA​A -3′
NADPH oxidase 4 (*NOX-4*): forward 5′-GCC​AGA​GTA​TCA​CTA​CCT​CCA​C-3′; reverse 5′- CTC​GGA​GGT​AAG​CCA​AGA​GTG​T -3′
Mitochondrial superoxide dismutase (*MnSOD*): forward 5′-CTG​GAC​AAA​CCT​CAG​CCC​TAA​C-3′; reverse 5′- AAC​CTG​AGC​CTT​GGA​CAC​CAA​C-3′
Catalase (*CAT*): forward 5′-GTG​CGG​AGA​TTC​AAC​ACT​GCC​A-3′; reverse 5′- CGG​CAA​TGT​TCT​CAC​ACA​GAC​G-3′

For *Adora1*, *adora2a*, and *2*β*m* genes, qPCR Primer Assays (Bio-Rad, Hercules, CA, United States) were ordered with the following Unique Assay IDs: *adora1*: qRnoCED0004330; *adora2a*: qRnoCED0004463; *2*β*m:* qRnoCED0056999. All primers were validated by Bio-Rad laboratories by analysing the amplification plot, melt peak and standard curve. At the end of all PCR cycles, a melt curve analysis was performed to confirm the specificity of primer annealing.

### 2.10 Statistical analysis

Results are expressed as mean ± standard error of the mean (SEM), with each experimental group including at least 4–6 replicates. The normality of distribution of the results was checked by using Shapiro-Wilk test. Group comparisons were conducted using unpaired T-test, one-way ANOVA followed by Dunnett’s or Šídák’s multiple comparison tests, as appropriate. All statistical analyses were carried out using GraphPad Prism (GraphPad 10.4.1 version, San Diego, CA, United States). Image acquisition and measurements were performed by a single investigator blinded to the experimental conditions. P values were two-tailed, and a threshold of P < 0.05 was considered statistically significant.

## 3 Results

### 3.1 Blood pressure and body weight

**FIGURE 1:**
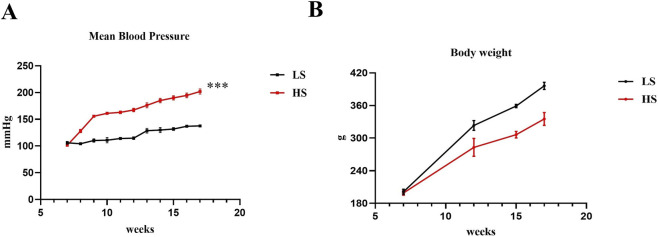
Time-course of mean blood pressure and body weight changes in the HFpEF model. **(A)** Mean blood pressure and **(B)** body weight measurements in Dahl salt-sensitive rats during a high-salt diet. Data are expressed as mean ± SEM. ***P < 0.001 vs. LS. Statistical significance was determined by unpaired T-test.

Dahl rats fed a high-salt diet exhibited a progressive increase in blood pressure, which became evident as early as the second week showed a ∼1.5-fold increase at 17 weeks of age compared to rats fed a low salt diet. This control animals maintained stable blood pressure over the 10-week study period (Figure 1A) (Cappetta et al., 2020). The increase in blood pressure in the HS group was associated with a decline in general health, as manifested by a smaller increase of body weight (Figure 1B).

### 3.2 Cardiac function and hypertrophy

**FIGURE 2:**
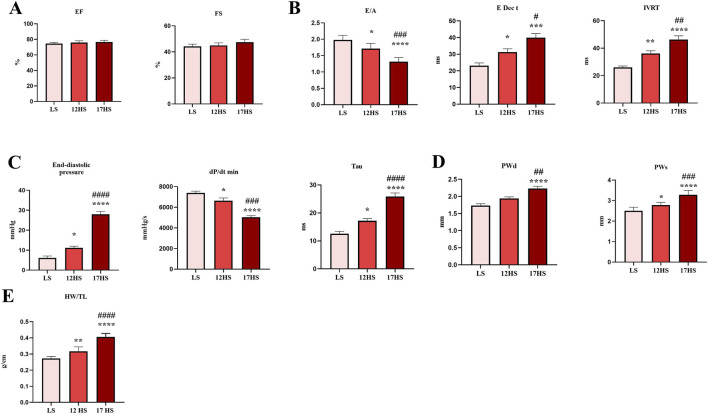
Echocardiographic and hemodynamic analysis during progression of HFpEF. **(A)** Echocardiographic measurements of ejection fraction (EF) and fractional shortening (FS). **(B)** Transmitral pulsed wave Doppler measurements of E wave and A wave ratio (E/A), E wave deceleration time (E Dec t), and isovolumetric relaxation time (IVRT). **(C)** Hemodynamic parameters of end-diastolic pressure, dP/dt min and time constant Tau. **(D)** Posterior wall thickness in diastole and systole. **(E)** Heart weight/tibial length (HW/TL) ratio. Data are expressed as mean ± SEM. *P < 0.05; **P < 0.01; ***P < 0.001; ****P < 0.0001 vs. LS; ^#^P < 0.05; ^##^P < 0.01; ^###^P < 0.001; ^####^P < 0.0001 vs. 12HS. Statistical significance was determined by one-way ANOVA followed by Sidak’s multiple comparison test.

To confirm the establishment of the HFpEF model, myocardial function was evaluated. At the end of experiment, echocardiography revealed preserved systolic function, as EF and FS remained unchanged among groups (Figure 2A). However, diastolic function, evaluated by echo-Doppler, revealed significant differences. Specifically, the 12HS group exhibited decrease E/A ratio, and prolonged E Dec t and IVRT, compared to LS. These abnormalities were further aggravated in 17HS group (∼0.7-fold decrease in E/A, ∼1.3-fold increase in E Dec time and ∼1.3-fold increase in IVRT compared to 12HS) (Figure 2B). In 17HS group, hemodynamic studies evidenced a similar pattern, as shown by elevated end-diastolic pressure (∼2.5-fold), decreased dP/dt min (∼0.8-fold), and prolonged relaxation indicated by time constant Tau (∼1.5-fold) (Figure 2C). Cardiac hypertrophy was evaluated by echocardiographic assessment evidencing a gradual increase in posterior wall thickness in HS groups, compared to LS animals. Also, heart weight/tibial length ratio progressively increased in HS group with a marked ∼1.3-fold increase observed in 17HS rats compared to 12HS (Figures 2D,E).

### 3.3 Histological analysis

**FIGURE 3:**
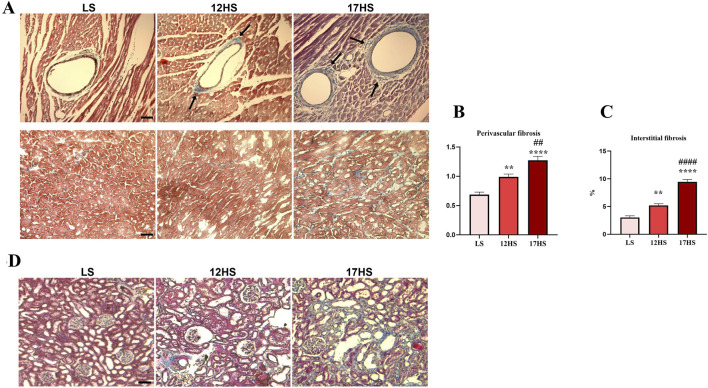
Progression of fibrosis in the heart and kidney of HFpEF model. **(A)** Representative images of LV sections with Masson’s trichrome staining showing perivascular (upper panels, arrows) and interstitial (lower panels) matrix deposition. Scale bars: 50 μm upper and lower panels. Quantification of collagen deposition in **(B)** perivascular and **(C)** interstitial LV areas. **(D)** Representative images of kidney sections with Masson’s trichrome staining showing the degree of renal fibrosis. Scale bar: 75 μm. Data are expressed as mean ± SEM. **P < 0.01; ****P < 0.0001 vs. LS; ^##^P < 0.01; ^####^P < 0.0001 vs. 12HS. Statistical significance was determined by one-way ANOVA followed by Sidak’s multiple comparison test.

As well known, oxidative stress and inflammation play a role in the development of fibrosis. In our HFpEF model, we observed a gradual increase in perivascular and interstitial fibrosis in the myocardium and kidney of HS animals, attributed to collagen buildup, as demonstrated by Masson’s trichrome staining (Figures 3A–D). Specifically, interstitial cardiac fibrosis increased by ∼1.7-fold in 12HS rats compared to controls and further progressed up to ∼3-fold increase in 17HS animals. Perivascular cardiac fibrosis also increased, showing a ∼1.5-fold increase in 12HS rats and ∼1.8-fold increase in 17HS animals compared to controls (Figures 3B,C).

**FIGURE 4:**
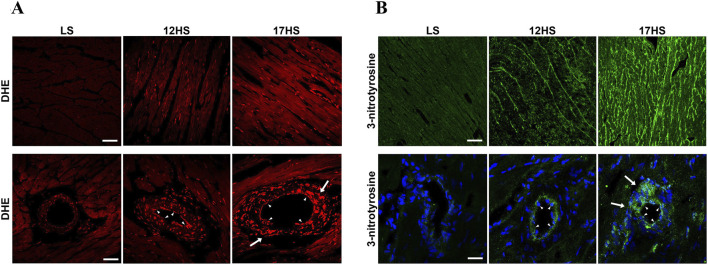
Progression of oxidative stress in the heart of HFpEF model. **(A)** Representative immunofluorescent showing dihydroethidium (DHE, red) in interstitial (upper panels) and perivascular (lower panels) LV areas. DHE-positive nuclei in the endothelium (arrowheads) and in the smooth muscle layer (arrows). Scale bars: 50 μm upper panels, 25 μm lower panels. **(B)** Representative immunofluorescent images showing 3-nitrotyrosine (green) in interstitial (upper panels) and perivascular (lower panels) LV areas. Nuclei were stained with DAPI (blue). Protein nitrosylation evidenced in the endothelium (arrowheads) and in the smooth muscle layer (arrows). Scale bars: 50 μm upper panels, 20 μm lower panels.

ROS were assessed by quantifying DHE fluorescence in cardiac sections from LS and HS rats. Hearts from the HS group displayed enhanced DHE fluorescence relative to LS controls, consistent with increased superoxide production (Figure 4A). In parallel, analysis of nitrative stress demonstrated elevated reactive nitrogen species in HS rats, as indicated by stronger 3-nitrotyrosine immunostaining at 12 and 17 weeks compared with LS animals (Figure 4B). The prominent presence of DHE and 3-nitrotyrosine in the endothelial lining of the coronary vasculature, indicated by arrowheads, as well as positive staining in the muscular layer of the vessel wall, indicating by arrows, strongly suggest vascular involvement in ROS production.

### 3.4 Proteome profiler

**FIGURE 5:**
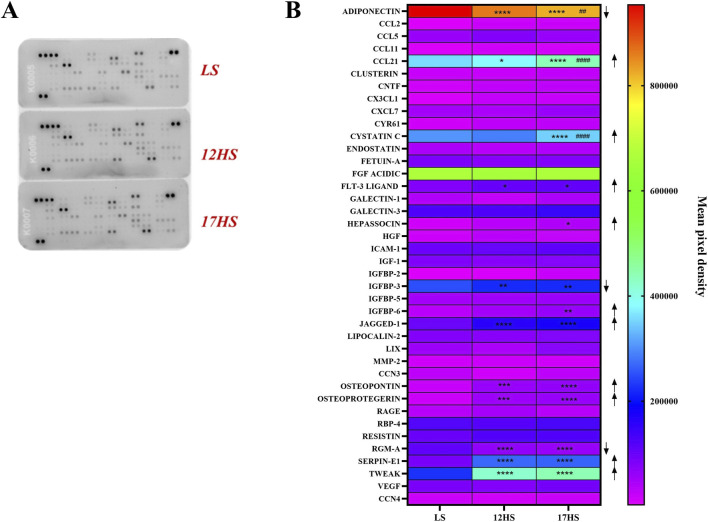
Proteome profiler analysis of cardiac cytokine and chemokine expression. **(A)** Representative proteome profiling array membranes. **(B)** Quantification of densitometric values presented as heatmap, expressed as INT/mm^2^. Data are expressed as mean ± SEM. Arrows indicate the direction of differential expression compared with the LS or 12HS groups (↑ upregulation, ↓ downregulation). *P < 0.05, **P < 0.01, ***P < 0.001, ****P < 0.0001 vs. LS, ^##^P < 0.01, ^####^P < 0.0001 vs. 12HS. Statistical significance was determined by two-way ANOVA followed by Dunnett’s or Sidak’s multiple comparisons test.

Proteome profiler analysis demonstrated distinct alterations in cardiac cytokine and chemokine expression between experimental groups (Figure 5A). In HS rats, CCL-21, Flt-3 ligand, Jagged-1, osteopontin, osteoprotegerin, Serpin-1, and TWEAK were significantly upregulated compared with controls (Figure 5B). Furthermore, cystatin C, hepassocin, and IGFBP-6 were selectively increased in animals that progressed to HFpEF. In contrast, adiponectin, IGFBP-3, and RGM-A levels were significantly reduced (Figure 5B). Together, these findings indicate activation of pro-inflammatory pathways under high-salt conditions, accompanied by downregulation of mediators with potential protective or anti-inflammatory roles.

### 3.5 Expression of enzymatic, transporter, and receptor components of the adenosine pathway and adenosine concentration in the heart

**FIGURE 6:**
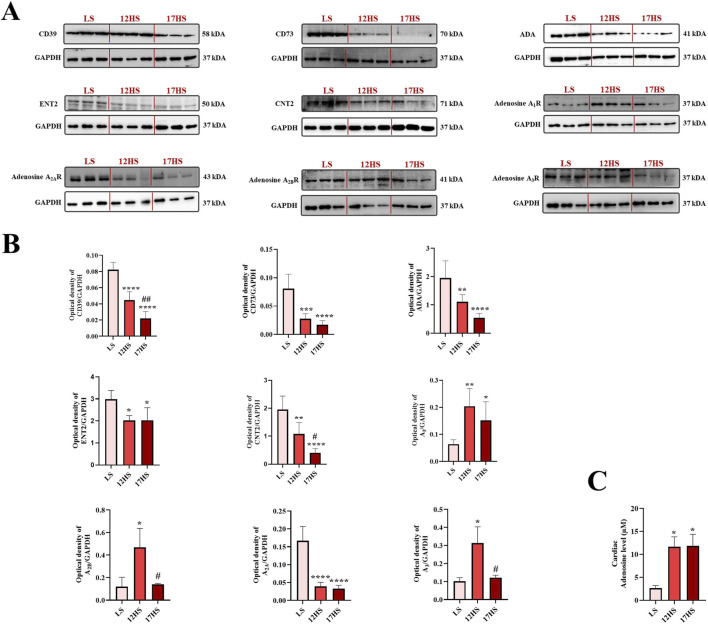
Expression of enzymatic/transporter/receptor components of the adenosine pathway and adenosine concentration in the heart. **(A)** Representative western blot images and **(B)** relative densitometric bar graphs. **(C)** Adenosine concentration in cardiac tissue measured by fluorometric assay. Data are expressed as mean ± SEM. *P < 0.05, **P < 0.01, ***P < 0.001, ****P < 0.0001 vs. LS; ^#^P < 0.05, p ^##^ < 0.01 vs. 12HS. Statistical significance was determined by one-way ANOVA followed by Sidak’s multiple comparisons test.

Expression of enzymatic, transporter and receptor components of the adenosine pathway was evaluated in cardiac tissue from Dahl rats by Western blotting. In 12HS rats, cardiac expression of the ecto-enzymes CD39 and CD73, which are key mediators of extracellular adenosine generation, was significantly reduced compared with LS controls, reaching ∼0.5-fold and ∼0.3-fold of control levels, respectively (Figures 6A,B). A further reduction was observed at 17 weeks, with CD39 and CD73 expression decreasing to ∼0.3-fold and ∼0.2-fold of LS levels, respectively (Figures 6A,B). In addition, expression of (ADA), the enzyme involved in adenosine degradation, was significantly downregulated, decreasing to ∼0.6-fold in 12HS rats and ∼0.3-fold in 17HS rats compared with LS controls (Figures 6A,B). Cardiac expression of the nucleoside transporters ENT2 (SLC29A2) and CNT2 (SLC28A2) was also significantly decreased in hypertensive high-salt–fed rats compared with controls, decreasing to ∼0.7-fold and ∼0.6-fold, respectively (Figures 6A,B). While ENT2 expression remained consistently reduced in animals that progressed to HFpEF, CNT2 expression showed a further significant decline at the HFpEF stage from ∼0.6-fold to ∼0.2-fold of LS levels (Figures 6A,B). These findings indicate an early impairment of both equilibrative and concentrative adenosine transport in response to high-salt–induced hypertension, with a progressive loss of CNT2-mediated nucleoside uptake accompanying HFpEF development. Among adenosine receptors, cardiac A_1_ receptor expression was significantly increased in HS-fed rats compared with LS controls, reaching ∼3-fold in 12HS rats and ∼2-fold in 17HS rats (Figures 6A,B). Conversely, A_2A_ receptor expression was significantly reduced in both HS groups, decreasing to ∼0.2-fold of LS levels (Figures 6A,B). Notably, A_2B_ and A_3_ receptor expression was significantly elevated in hypertensive animals increasing to ∼4-fold and ∼3-fold, respectively, but returned to control levels in rats that progressed to HFpEF (Figures 6A,B). Adenosine levels measured in cardiac tissue were significantly increased in both the 12HS and 17HS experimental groups compared to the LS control group (Figure 6C). No significant differences were observed between the two HS groups, suggesting that both early and late stages of the experimental model similarly elevate cardiac adenosine concentrations.

### 3.6 Expression/Distribution profile of adenosine receptors in kidney

**FIGURE 7:**
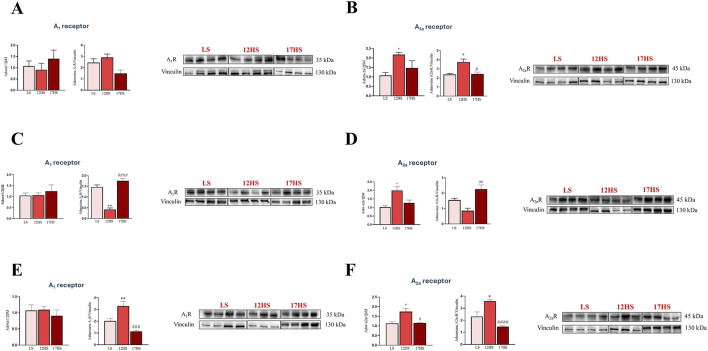
Gene and protein expression and relative bands of A_1_ and A_2A_ adenosine receptors in the kidney. **(A,B)**, cortex; **(C,D)** outer medulla; **(E,F)** inner medulla. The histograms show the relative content of transcript levels (left panels), densitometric protein analysis normalized to vinculin (right panels) and western blot bands. Data are expressed as mean ± SEM. *P < 0.05, **P < 0.001 vs. LS; ^#^P < 0.05, ^##^P < 0.001, ^###^P < 0.0001, ^####^P < 0.00001 vs. 12HS. Statistical significance was determined by one-way ANOVA followed by Sidak’s multiple comparisons test.

To investigate the potential role of adenosine receptors in renal dysfunction associated with HF, we assessed the gene and protein expression of A_1_R and A_2A_R, the adenosine receptors mainly involved in salt-sensitive hypertension (Uehara et al., 1995; Zou et al., 1999; Dobrowolski et al., 2007; Denton, 2018), in the cortex, outer medulla, and inner medulla of salt-induced hypertensive rats at 12 and 17-weeks, alongside control groups. Gene expression analysis revealed no significant changes in A_1_R mRNA levels across the three kidney regions (Figures 7A,E). However, A_1_R protein expression was significantly reduced to ∼0.3-fold in the outer medulla and increased ∼1.6-fold in the inner medulla of HS animals at 12 weeks compared to LS animals (Figures 7C,E). Notably, these alterations returned to control levels in 17HS rats that had progressed to HFpEF (Figures 7C,E). We also observed a significant upregulation of A_2A_R at both the gene and protein levels in the cortex and inner medulla of 12-week HS rats. In the cortex, A_2A_R expression increased by ∼3.6-fold at the gene level and ∼1.4-fold at the protein level, whereas in the inner medulla it increased by ∼1.22-fold at the gene level and ∼2.3-fold at the protein level. These changes returned toward control levels in 17-week HS rats (Figures 7B,F). In contrast, in the outer medulla, the significant increase in *Ador-a2a* mRNA levels in 12HS rats (∼1.54-fold) was followed by an increase in protein levels in 17HS rats (∼3.3-fold) compared to 12HS rats (Figure 7D).

### 3.7 Evaluation of endothelial response in HFpEF and HCMECs

**FIGURE 8:**
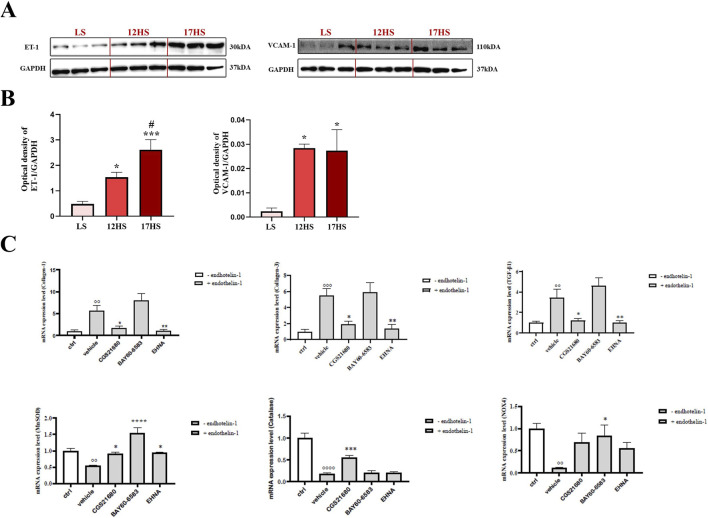
Expression of endothelin-1 and VCAM-1 in cardiac tissue and modulation by A_2_ receptor agonists and EHNA in HCMECs. **(A)** Representative western blot images and **(B)** relative densitometric bar graphs. *P < 0.05, ***P < 0.001 vs. LS; ^#^P < 0.05 vs. 12HS. **(C)** Relative mRNA expression levels of *COL1, COL3, TGFB1, NOX-4, MnSOD and CAT* in HCMECs following stimulation with endothelin-1 in the absence or presence of CGS21680, BAY60-6583, or EH. *P < 0.05, **P < 0.01, ***P < 0.001, ****P < 0.0001 vs. vehicle; °°P < 0.05, °°°P < 0.001, °°°°P < 0.0001 vs. ctrl. Data are expressed as mean ± SEM. Statistical significance was determined by one-way ANOVA followed by Sidak’s multiple comparisons test.

In the hearts of HS rats, endothelin-1 and the endothelial activation marker VCAM-1 were significantly upregulated (Figures 8A,B), pointing to enhanced endothelial activation together with vasoconstrictive and pro-fibrotic signaling typical of HFpEF. To further explore these mechanisms, HCMECs were exposed to ET-1 and treated either with selective A_2A_ and A_2B_ receptor agonists or with EHNA to enhance endogenous adenosine signaling, given the established role of adenosine and these receptor subtypes in regulating vascular tone, permeability, and inflammation.

Stimulation with ET-1 significantly upregulated the expression of *COL-1* (∼5-fold)*, COL-3* (∼5-fold), and *TGF-β1* (∼3-fold) in HCMECs relative to untreated control cells. Notably, treatment with the A_2A_ receptor agonist CGS21680 (10 µM) significantly attenuated this ET-1–induced upregulation reducing COL-1, COL-3, and TGF-β1 expression to ∼1.7-fold, ∼1.9-fold, and ∼1.2-fold, respectively (Figure 8C). Similarly, inhibition of adenosine deaminase with EHNA (10 µM) markedly blunted ET-1–mediated fibrotic signaling, lowering expression levels to ∼1-fold for COL-1, COL-3 and TGF-β1 at gene level (Figure 8C). These results indicate a protective, anti-fibrotic effect mediated by A_2A_ receptor activation or enhanced adenosine signaling. In contrast, the A_2B_ receptor agonist BAY60-6583 (10 µM) did not significantly alter the expression of these ECM-related genes (Figure 8C), suggesting a limited role for A_2B_ receptors in regulating ET-1–driven profibrotic responses in this context.

Regarding oxidative stress markers, ET-1 exposure significantly reduced MnSOD expression to approximately ∼0.5-fold relative to untreated control cells. *MnSOD.* This effect was reversed by treatment with CGS21680, BAY60-6583, and EHNA, each of which significantly increased *MnSOD* gene expression reaching ∼0.9-, ∼1.5-, and ∼0.9-fold, respectively (Figure 8C). However, distinct receptor-specific effects were observed for other antioxidant genes: CGS21680 uniquely upregulated *CAT* expression, while only BAY60-6583 increased *NOX-4* expression (Figure 8C).

## 4 Discussion

The present study provides new evidence that impaired adenosine signaling contributes to the pathophysiology of HFpEF. At the cellular level, selective modulation of adenosine receptors and metabolism exerted protective effects on cardiac endothelial cells exposed to ET-1. Using the Dahl salt-sensitive rat model, which reproduces the hypertensive and fibrotic phenotype typical of human HFpEF (De Angelis et al., 2016), we demonstrated that the cardiac expression of CD39 and CD73, two key ecto-enzymes that generate extracellular adenosine, was markedly reduced in HS rats. This suggests an impairment in adenosine production. In parallel, ADA, the enzyme responsible for adenosine degradation, was also downregulated, indicating a combined alteration in adenosine turnover that could disrupt signaling and worsen cardiac dysfunction.

Unexpectedly, despite these enzymatic reductions, direct quantification revealed increased myocardial adenosine levels in both early hypertensive and HFpEF-stage animals. This apparent paradox likely reflects a compensatory response to chronic stressors such as hypoxia, oxidative stress, and inflammation, hallmarks of hypertensive heart disease and HFpEF (Khodadadi et al., 2014).

Under these conditions, intracellular adenosine accumulation is driven by enhanced ATP catabolism and impaired clearance rather than increased enzymatic production. In line with this interpretation, reduced ADA expression limits intracellular adenosine degradation, thereby favouring its accumulation (Mörschl et al., 2008). Consistent with previous reports showing increased tissue adenosine levels under hypoxic or ischemic conditions despite reduced expression of metabolic enzymes, these findings support the concept that adenosine buildup in HFpEF is primarily driven by cellular stress rather than enzymatic control alone.

In addition to enzymatic regulation, extracellular adenosine availability is critically shaped by membrane transport mechanisms. Both ENT2 and CNT2 were already downregulated at the hypertensive stage, indicating that impairment of nucleoside transport occurs early in response to high-salt–induced cardiovascular stress. While ENT2 expression remained persistently reduced in HFpEF, CNT2 was further significantly downregulated at the HFpEF stage, suggesting a selective and progressive loss of sodium-dependent adenosine uptake during disease progression. Given the established role of CNT2 in limiting extracellular adenosine availability through intracellular clearance (Gray et al., 2004; Pastor-Anglada and Pérez-Torras, 2018a; Pastor-Anglada and Pérez-Torras, 2018b), its progressive reduction may represent a maladaptive alteration that sustains myocardial adenosine signaling independently of enzymatic regulation. Moreover, the concomitant downregulation of nucleoside transporters likely impairs adenosine reuptake and intracellular redistribution, thereby contributing to persistently elevated myocardial adenosine levels under chronic stress conditions (Eltzschig et al., 2012; Boswell-Casteel and Hays, 2017).

At the cardiac level, HS rats exhibited significantly increased A_1_ receptor expression and reduced A_2A_ receptor expression. A_1_ receptor upregulation may represent a compensatory attempt to preserve cardiac protection by inhibiting adenylate cyclase and reducing heart rate, while A_2A_ receptor downregulation likely contributes to maladaptive inflammation and oxidative stress (Asakura et al., 2007; Boknik et al., 2021). Interestingly, A_2B_ and A_3_ receptors were transiently upregulated in hypertensive rats but normalized in those progressing to HFpEF, despite continued high-salt exposure, suggesting that receptor remodeling is not solely driven by salt intake. Although high dietary NaCl has been shown to modulate adenosine metabolism and receptor expression independently of overt cardiovascular disease, including in salt-sensitive models (Zou et al., 1999; Rajagopal and Pao, 2010; Jackson et al., 2018), the observed temporal pattern supports a disease-stage–dependent, adaptive-to-maladaptive shift in adenosine signaling as hypertension evolves into overt HFpEF under chronic renal and cardiac stress. Nevertheless, the contribution of salt exposure remains a limitation that warrants further investigation in salt-independent HFpEF models.

Another important limitation of the present study is that adenosine receptor expression was assessed in left ventricular homogenates, reflecting a composite signal from cardiomyocytes, endothelial cells, fibroblasts, and immune cells, and therefore does not allow cell-specific resolution. Adenosine receptors are differentially expressed across these cell populations, and their functional roles are strongly cell-type dependent (Fredholm et al., 2011; Eltzschig et al., 2012). Consequently, the myocardial adenosine receptor alterations observed here likely reflect an integrated multicellular response rather than cell-specific regulation. Future studies employing spatially resolved analyses such as immunofluorescence, *in situ* hybridization, or single-cell transcriptomics will be essential to determine whether adenosine receptor–based interventions should be directed systemically or tailored to specific cardiac cell populations to maximize efficacy while minimizing off-target effects.

Renal analysis revealed time-dependent and region-specific modulation of A_1_ and A_2A_ receptors. As in the heart, these changes normalized in advanced disease stages (17HS), indicating an adaptive transition during HFpEF development. Notably, A_2A_ receptor protein was markedly elevated in the outer medulla of 17HS rats, consistent with its role in regulating sodium chloride balance via the cAMP/PKA pathway (Oyarzún et al., 2017). We focused on A_1_ and A_2A_ receptors in cortex, outer, and inner medulla due to their central roles in renal hemodynamics, tubuloglomerular feedback, and blood pressure control processes that are directly pertinent to salt-sensitive hypertension and the pathophysiology leading to HFpEF (Welch, 2015; Zaika et al., 2020). Previous studies in DS rats have implicated altered A_1_ signaling and impaired A_2A_-mediated protective pathways in salt-induced hypertension (Uehara et al., 1995; Zou et al., 1999; Dobrowolski et al., 2007; Denton, 2018). In contrast, A_2B_ and A_3_ receptors, typically expressed at low levels in healthy kidneys, become prominent only during advanced inflammation or fibrosis (Feoktistov and Biaggioni, 2011). Our findings thus highlight the relevance of A_1_ and A_2A_ receptors in early renal dysfunction associated with HFpEF, while suggesting that A_2B_ and A_3_ pathways may emerge at later stages.

Our data point to a profound imbalance in adenosine signaling in HFpEF, aligning with its known roles in endothelial dysfunction, inflammation, and fibrosis (Eltzschig et al., 2012; Borea et al., 2016). Initially, elevated adenosine may confer cardioprotection via A_1_ and A_2A_ receptors; however, chronic accumulation coupled with receptor dysregulation, especially A_2A_ downregulation and transient A_2B_/A_3_ upregulation, may shift signaling toward maladaptation. In later HFpEF stages, normalization of A_2B_/A_3_ expression could signify loss of compensatory capacity, promoting fibrosis, oxidative stress, and vascular dysfunction (Dubey et al., 1998; Nishat et al., 2016). Similar findings in failing human myocardium, where A_2A_, A_2B_, A_3_, and ADA were reduced while adenosine levels were elevated (Asakura et al., 2007), support a conserved pathogenic mechanism. These findings underscore the complexity of adenosine signaling in the heart and suggest that total adenosine levels alone are not indicative of beneficial signaling, particularly when receptor expression and downstream pathways are altered in disease states.

Histological and proteomic analyses confirmed enhanced oxidative stress and fibrosis in HS rats, consistent with prior evidence of reactive oxygen and nitrogen species driving HFpEF progression (Tanaka and Shimizu, 2012; Tojo et al., 2002). Cytokine profiling revealed upregulation of pro-inflammatory mediators including CCL-21, Flt-3 ligand, Jagged-1, osteopontin, osteoprotegerin, Serpin-1, and TWEAK all linked to endothelial dysfunction and fibrotic remodeling (Scatena et al., 2007; Wollert et al., 2012; Unudurthi et al., 2020). In contrast, HFpEF-stage animals exhibited increases in cystatin C, hepassocin, and IGFBP-6, reflecting activation of metabolic and stress pathways (Xie et al., 2010), alongside loss of protective molecules such as adiponectin, IGFBP-3, and RGM-A.

Endothelial activation and impaired endothelial signaling have been widely recognized as key contributors to diastolic dysfunction and myocardial remodeling (Cappetta et al., 2020). In our study, although we did not specifically dissect the contributions of different cardiac and extracardiac cell types to the endothelin pool, the observed increase in ET-1 and VCAM-1 expression in left ventricular homogenates strongly suggests activation of the endothelial compartment, which represents a major source of ET-1. While other cardiac cell types may also contribute, the overall evidence supports the notion that endothelial dysfunction is a central, albeit not exclusive, driver of HFpEF in our model. Consistent with this interpretation, elevated ET-1 and VCAM-1 levels confirm the presence of endothelial activation and microvascular inflammation (Paulus and Tschope, 2013). In HCMECs, ET-1 induced profibrotic genes (*COL-1, COL-3, TGF-β1*) and oxidative imbalance. A_2A_ receptor activation or ADA inhibition significantly attenuated these effects, whereas A_2B_ receptor activation did not, supporting receptor-specific anti-fibrotic actions via A_2A_ signaling (Scuruchi et al., 2023; Ryzhov et al., 2014). Moreover, both A_2A_ and A_2B_ activation, as well as ADA inhibition, restored MnSOD expression suppressed by ET-1, indicating improved antioxidant defenses, though only A_2A_ stimulation increased *CAT* expression, suggesting a broader antioxidant response through A_2A_-mediated pathways (Son et al., 2003; Castro et al., 2020).

Interestingly, A_2B_ receptor activation enhanced *NOX-4* expression, which, though traditionally linked to ROS production, can also mediate cytoprotective signaling and angiogenesis under specific contexts (Zhang et al., 2010; Wang et al., 2014). Thus, A_2B_ signaling may serve adaptive redox roles depending on disease stage. Collectively, these findings illustrate a receptor-specific regulation of oxidative balance: A_2A_ primarily enhancing antioxidant defense, and A_2B_ modulating redox signaling that can be either adaptive or deleterious.

The functional impact of adenosine receptor subtypes in the heart is highly context dependent. A_1_ receptors generally confer cardioprotection and limit stress but may also contribute to fibrosis under certain stimuli (Lankford et al., 2006; Funakoshi et al., 2006). A_2B_ receptors display complex, time-dependent effects in post-infarction remodeling, mediating both adaptive and maladaptive outcomes depending on timing and cell type (Wakeno et al., 2006; Zhang et al., 2014). Thus, adenosine receptor signaling is not only receptor-specific but also dynamically shaped by disease stage, cell context, and systemic conditions, emphasizing the potential for precision therapeutic approaches.

In this context, it is important to acknowledge that HFpEF treatment has evolved in recent years toward recognizing endothelial and microvascular dysfunction as central contributors to disease development, positioning the endothelium as a specific and mechanistically relevant therapeutic target. SGLT2i have emerged as disease-modifying agents in HFpEF and are now recommended by the 2023 ESC Focused Update (Class I, Level A) based on EMPEROR-Preserved and DELIVER trials, with benefits driven primarily by reductions in HF hospitalizations (McDonagh et al., 2023).

With growing evidence that these effects are mediated, at least in part, by pleiotropic vascular actions, experimental studies indicate that SGLT2 inhibition improves endothelial function, attenuates oxidative stress, and restores microvascular homeostasis (Góes-Santos et al., 2025; Walczak et al., 2025; Tarnawska et al., 2025; Parzuchowska et al., 2025). Of note, it has been previously demonstrated that SGLT2i counteracted endothelial dysfunction in HFpEF patients (Walczak et al., 2025). All these mechanisms closely intersect with the adenosine-dependent pathways identified in the present study.

In parallel, emerging evidence supports additional phenotype-directed strategies in selected HFpEF populations, including mineralocorticoid receptor antagonists (MRAs) and incretin-based therapies. MRAs may provide benefit in selected HFpEF patients, particularly with respect to HF hospitalizations and specific clinical profiles (Ferreira et al., 2024). GLP-1 receptor agonists have recently shown clinically meaningful improvements in symptoms, quality of life, and functional capacity in obesity-related HFpEF, highlighting a promising, primarily symptom- and phenotype-oriented approach rather than established hard-outcome modification at this stage (Kosiborod et al., 2023).

It should also be noted that these agents are used on top of optimized background management, particularly diuretics for congestion and comprehensive treatment of comorbidities (hypertension, obesity, diabetes), underscoring the need for integrated strategies that combine systemic hemodynamic control with modulation of endothelial, metabolic, and microvascular dysfunction, processes that are highly relevant to the adenosine-dependent mechanisms highlighted in the present study.

Although clinical trials targeting adenosine receptors in HFpEF have shown limited success (Shah et al., 2019), our results suggest that selective activation of A_2A_ receptors, potentially combined with strategies to increase extracellular adenosine, could restore endothelial function and reduce oxidative stress. The discrepancy between preclinical and clinical outcomes highlights HFpEF’s multifactorial nature, where comorbidities and systemic inflammation complicate pharmacological responses. Nonetheless, adenosine signaling remains a promising therapeutic axis linking the heart and kidney. Future investigations should determine whether endothelial protection via A_2A_ activation translates into improved cardiac structure and performance *in vivo*. Given HFpEF’s complex pathogenesis, combinatorial strategies integrating adenosine modulation with antifibrotic or antioxidant therapies may be required. Further studies should clarify how adenosine signaling mediates the transition from hypertension to HFpEF and whether targeting this pathway can yield tangible clinical benefits.

## Data Availability

The original contributions presented in the study are included in the article/Supplementary Material, further inquiries can be directed to the corresponding authors.
