# Supplementary material for: Impaired adenosine pathways in HFpEF: insights into cardiorenal alterations and endothelial responses
Source: Front Pharmacol. 2026 Feb 11;17:1720123. doi: 10.3389/fphar.2026.1720123 (PMC12932206; doi:10.3389/fphar.2026.1720123)
Supplement: Supplementary file 1 [file DataSheet1.pdf]

Cardiac expression of CD73

1A 1B 1C

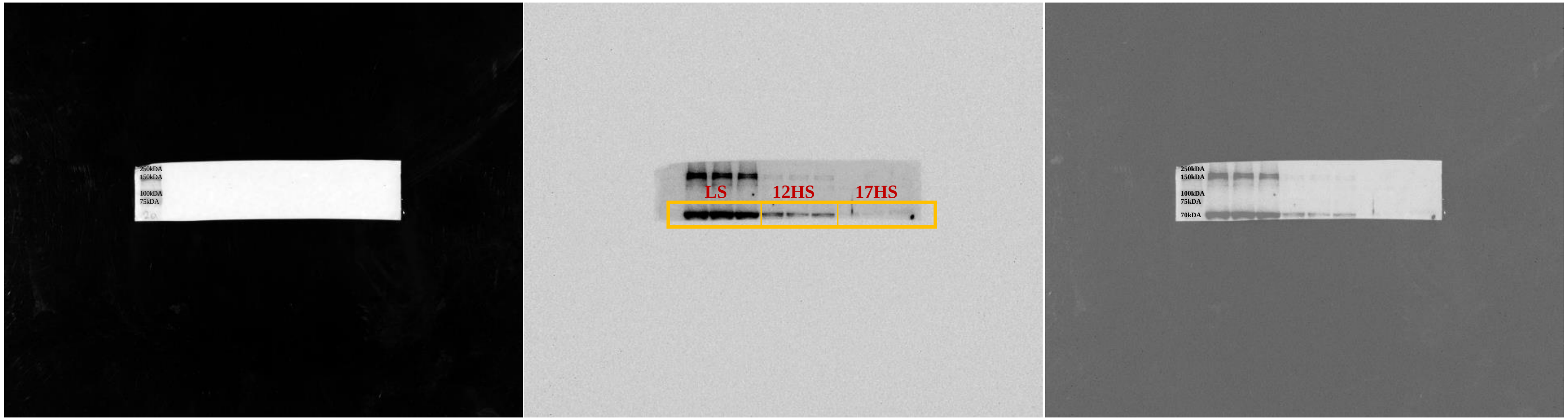

Fig.1 Original blot images of cardiac CD73 expression. *(Novus, NBP1-85740, 1:1000)* (Figure 1A) Colorimetric; (Figure 1B) Chemiluminescence; (Figure 1C) Merge Yellow boxes indicate the regions used for signal quantification.

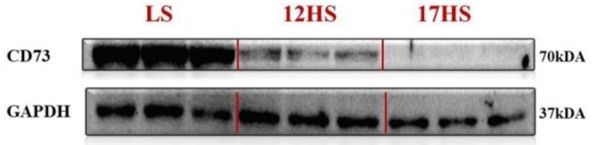

2A 2B 2C

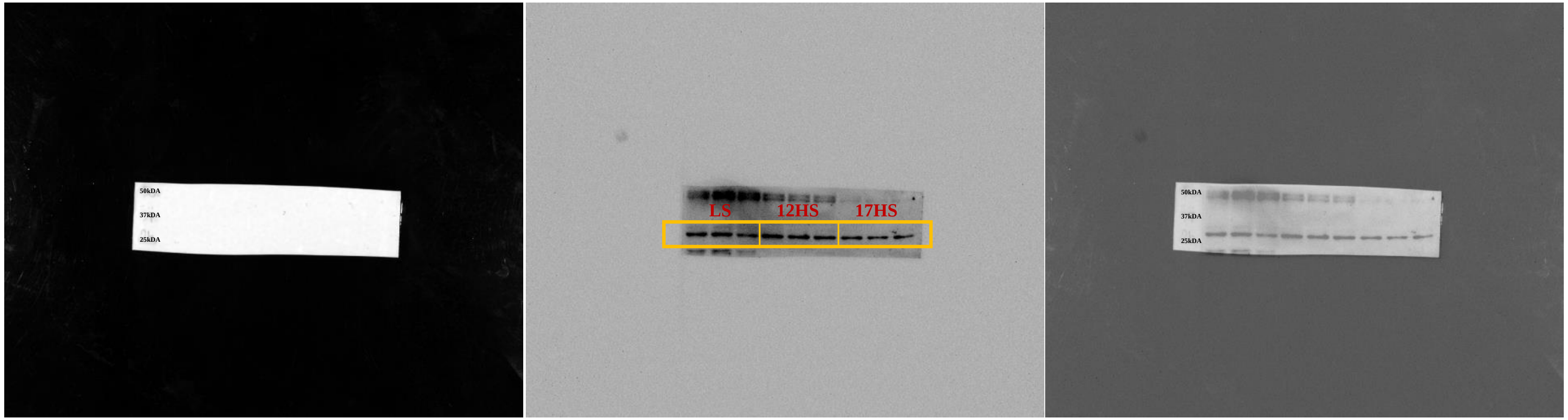

Fig.2 Original blot images of cardiac GAPDH expression. *(Sigma, G8795-100UL, 1:10000).* (Figure 2A) Colorimetric; (Figure 2B) Chemiluminescence; (Figure 2C) Merge Yellow boxes indicate the regions used for signal quantification.

Cardiac expression of CD39

3A

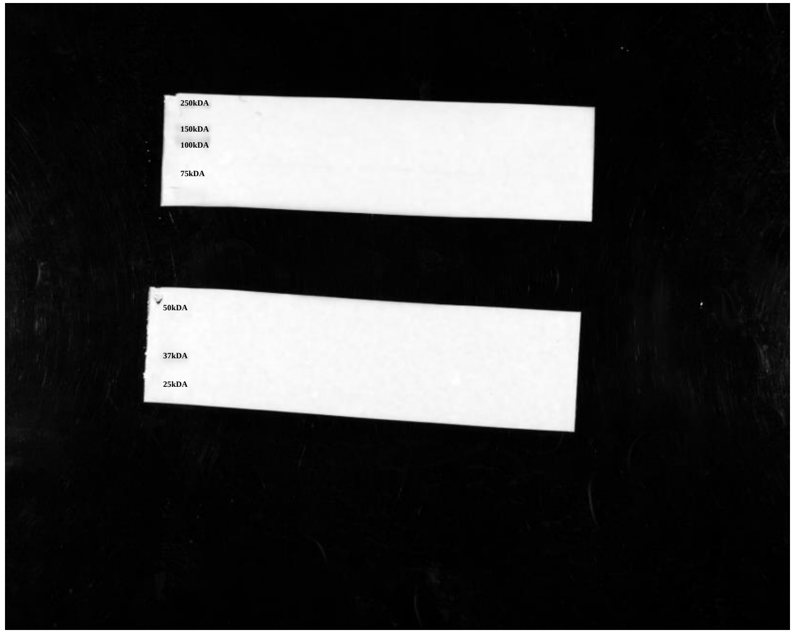

3B

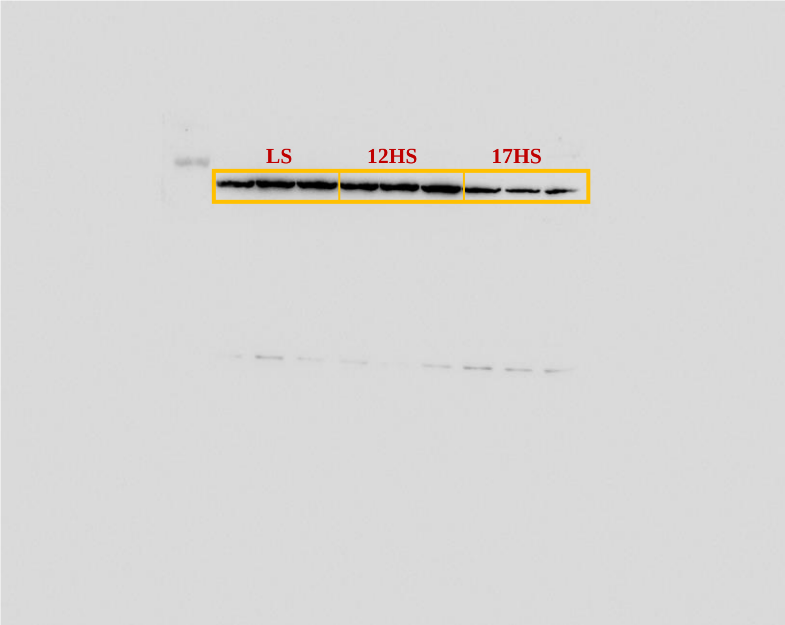

3D

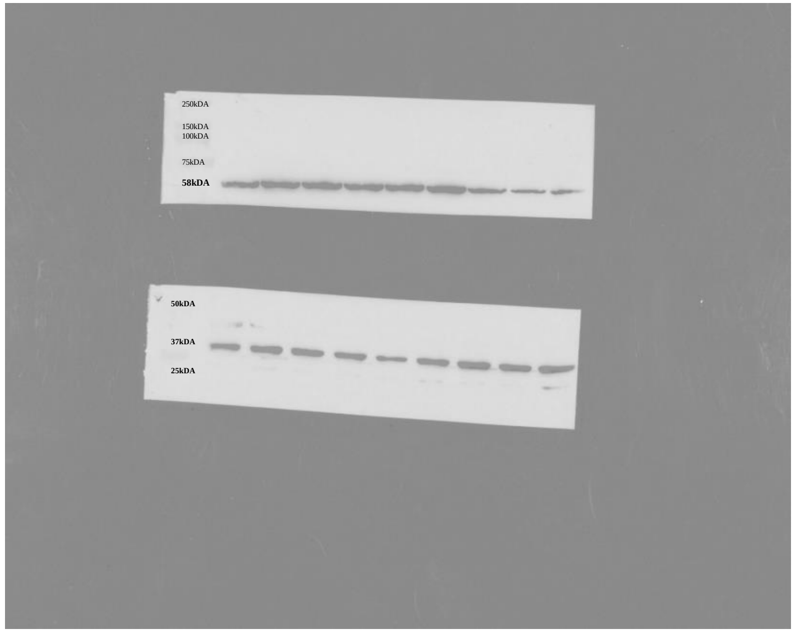

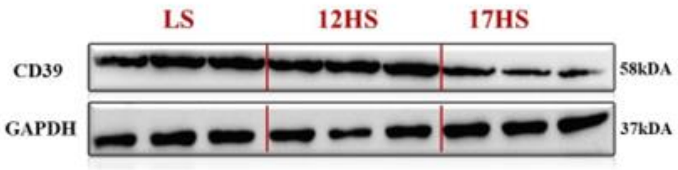

3C

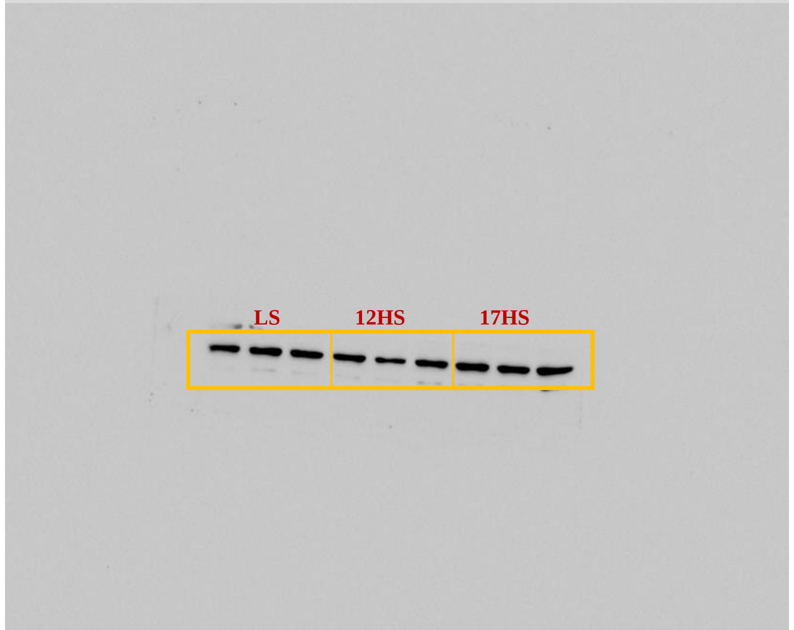

Fig.3 Original blot images of cardiac CD39 expression. *(Novus, NBP2-25223, 1:1000)* and its GAPDH *(Sigma, G8795-100UL, 1:10000)*
(Figure 3A) Colorimetric; (Figure 3B) Chemiluminescence of CD39; (Figure 3C) Chemiluminescence of GAPDH; (Figure 3D) Merge
Yellow boxes indicate the regions used for signal quantification.

Cardiac expression of ADA

4A **4B** **4C**

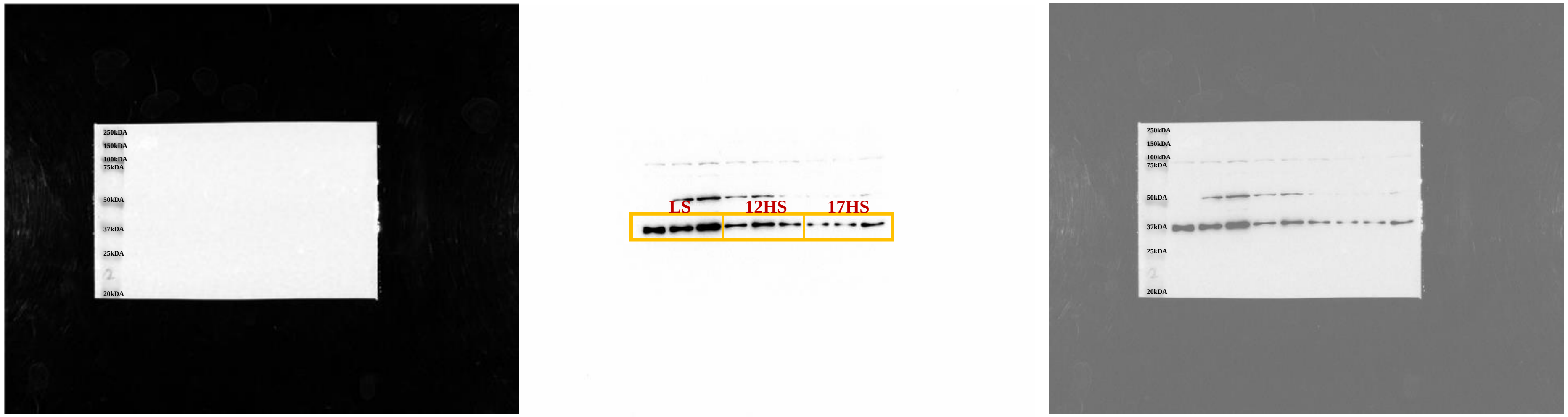

Fig.4 Original blot images of cardiac ADA expression. *(Novus, NBP1-87404, 1:500)* (Figure 4A) Colorimetric; (Figure 4B) Chemiluminescence; (Figure 4C) Merge Yellow boxes indicate the regions used for signal quantification.

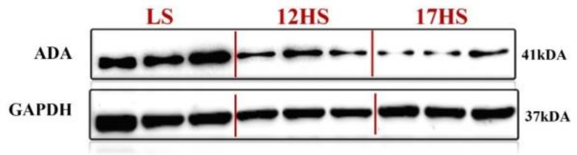

5A **5B** **5C**

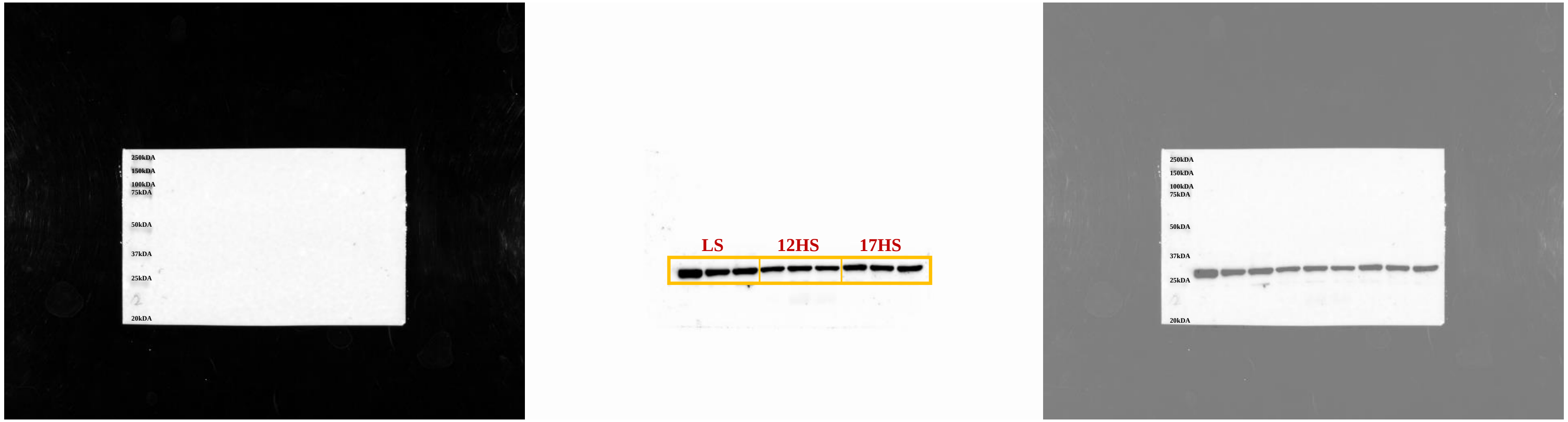

Fig.5 Original blot images of cardiac GAPDH expression. *(Sigma, G8795-100UL, 1:10000).* (Figure 5A) Colorimetric; (Figure 5B) Chemiluminescence; (Figure 5C) Merge Yellow boxes indicate the regions used for signal quantification.

Cardiac expression of A_1R

6A 6B 6C

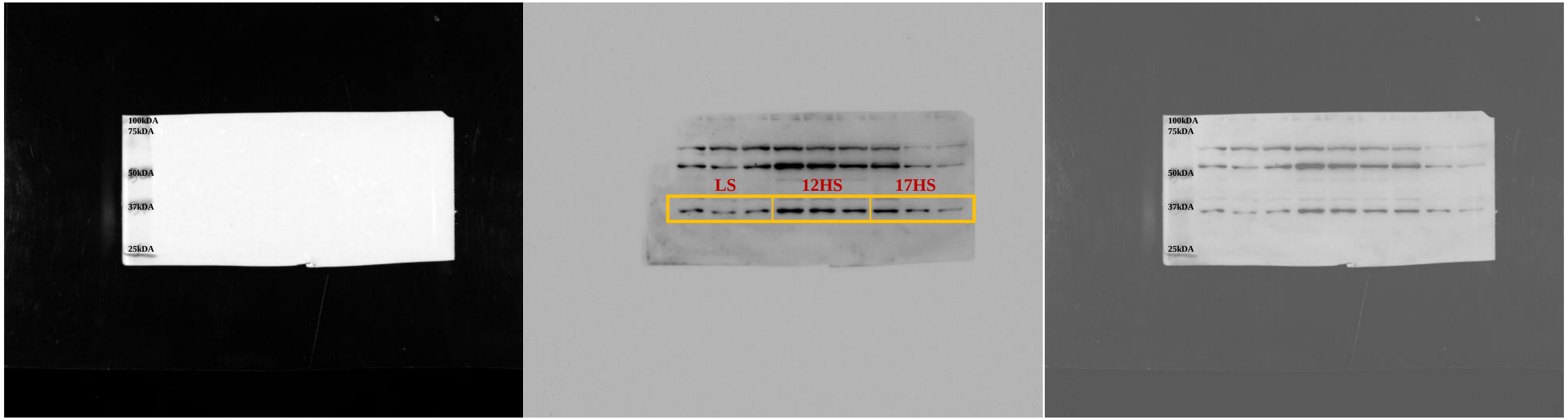

Fig.6 Original blot images of cardiac A_1R expression. *(Novus, NB300-549, 1:1000).* (Figure 6A) Colorimetric; (Figure 6B) Chemiluminescence; (Figure 6C) Merge
Yellow boxes indicate the regions used for signal quantification.

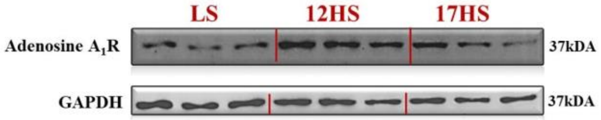

7A 7B 7C

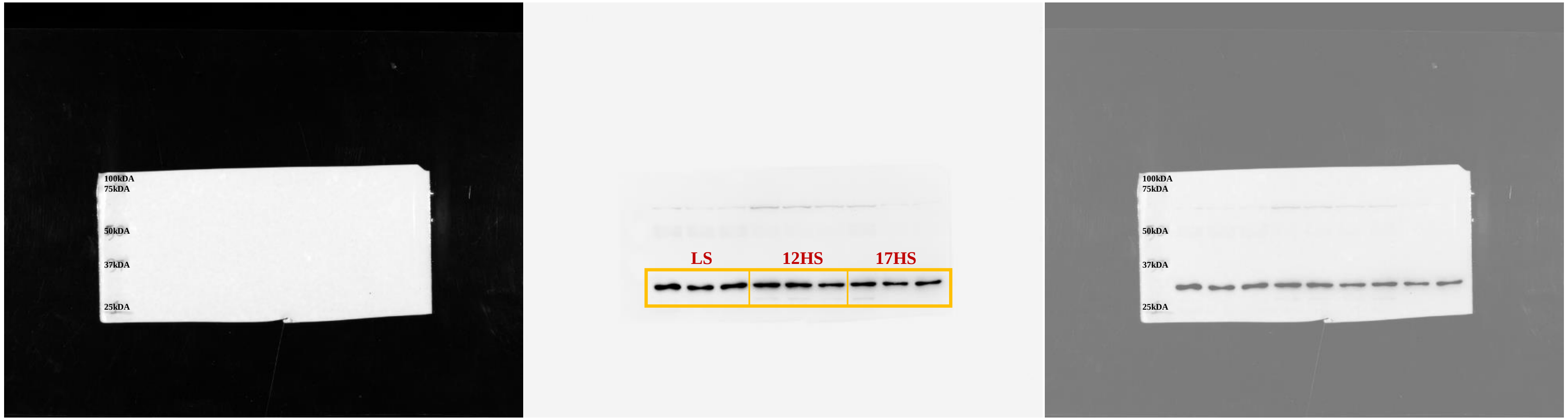

Fig.7 Original blot images of cardiac GAPDH expression. *(Sigma, G8795-100UL, 1:10000).* (Figure 7A) Colorimetric; (Figure 7B) Chemiluminescence; (Figure 7C) Merge
Yellow boxes indicate the regions used for signal quantification.

Cardiac expression of $A_{2A}R$

8A 8B 8C

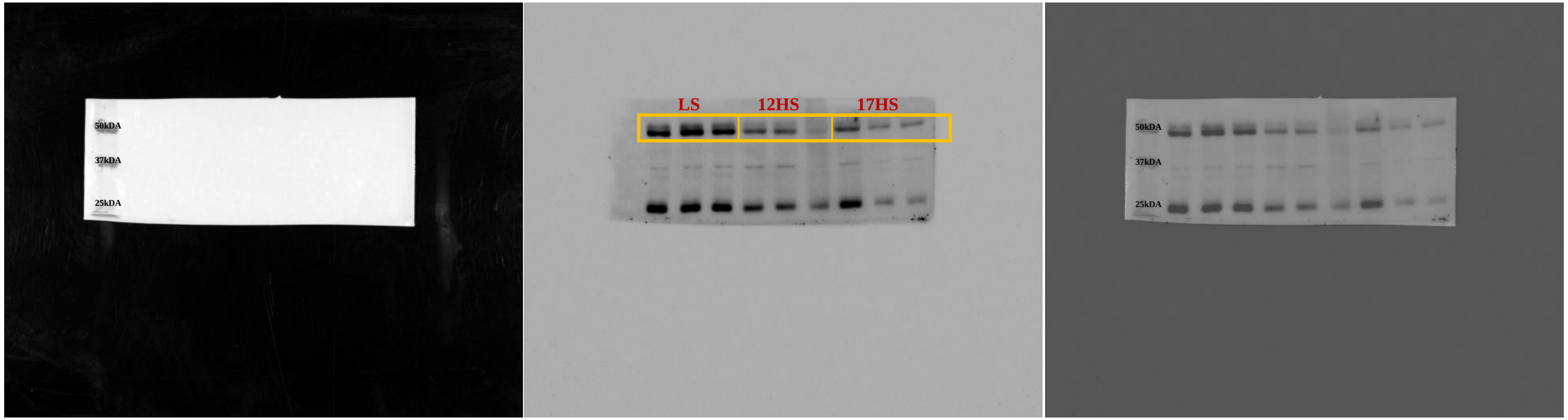

Fig.8 Original blot images of cardiac $A_{2A}R$ expression. *(Novus, NBP1- 39474, 1:1000).* (Figure 8A) Colorimetric; (Figure 8B) Chemiluminescence; (Figure 8C) Merge
Yellow boxes indicate the regions used for signal quantification.

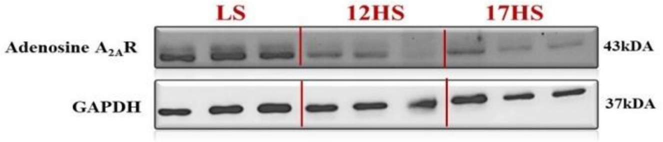

9A 9B 9C

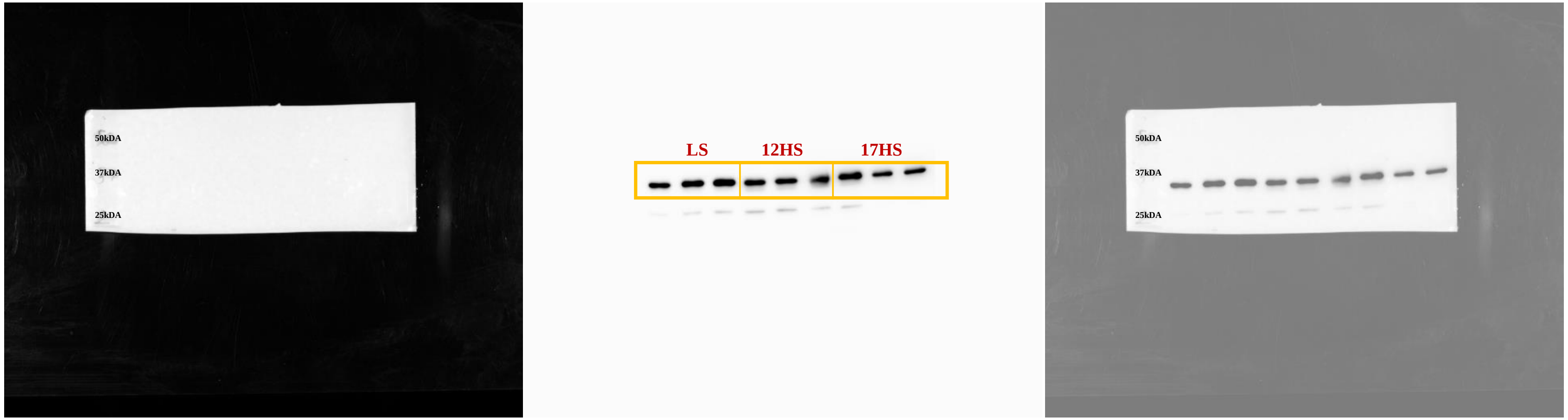

Fig.9 Original blot images of cardiac GAPDH expression. *(Sigma, G8795-100UL, 1:10000).* (Figure 9A) Colorimetric; (Figure 9B) Chemiluminescence; (Figure 9C) Merge
Yellow boxes indicate the regions used for signal quantification.

Cardiac expression of $A_{2B}R$

10A **10B** **10C**

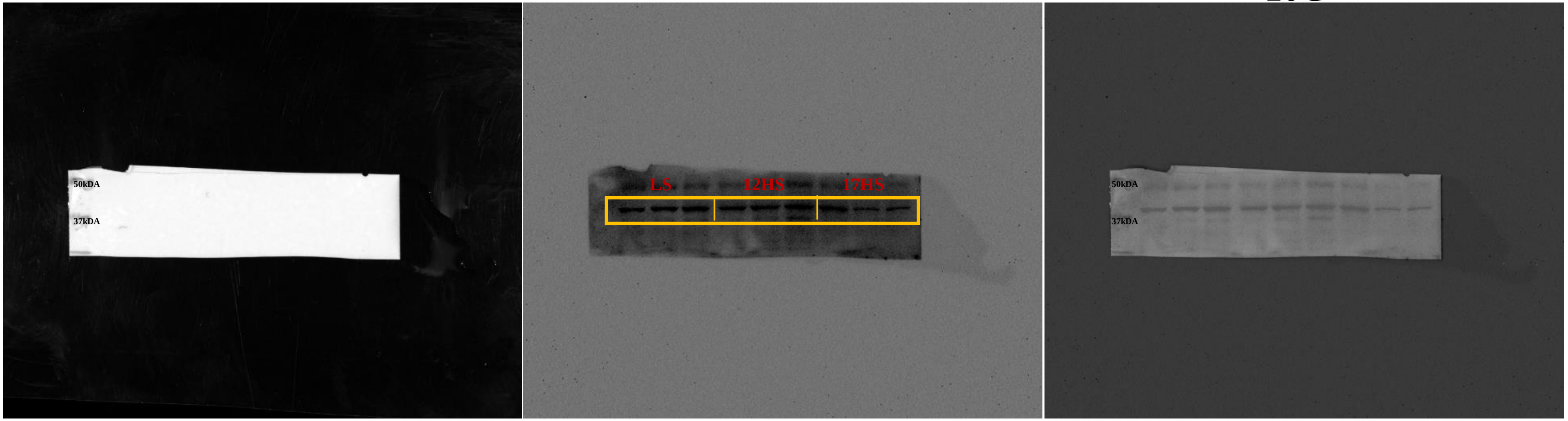

Fig.10 Original blot images of cardiac $A_{2B}R$ expression. *(Novus, NBP2-41312, 1:2000).* (Figure 10A) Colorimetric; (Figure 10B) Chemiluminescence; (Figure 10C) Merge Yellow boxes indicate the regions used for signal quantification.

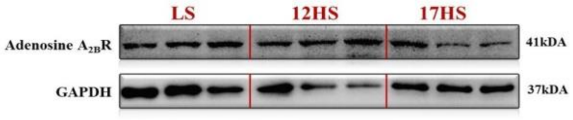

11A **11B** **11C**

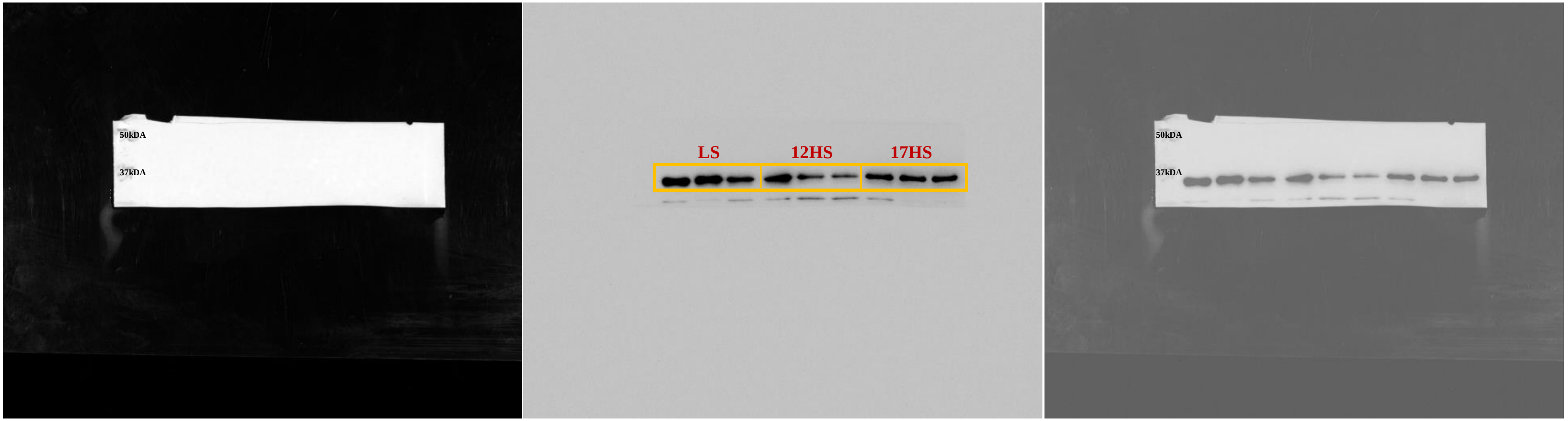

Fig.11 Original blot images of cardiac GAPDH expression. *(Sigma, G8795-100UL, 1:10000).* (Figure 11A) Colorimetric; (Figure 11B) Chemiluminescence; (Figure 11C) Merge Yellow boxes indicate the regions used for signal quantification.

Cardiac expression of A_3R

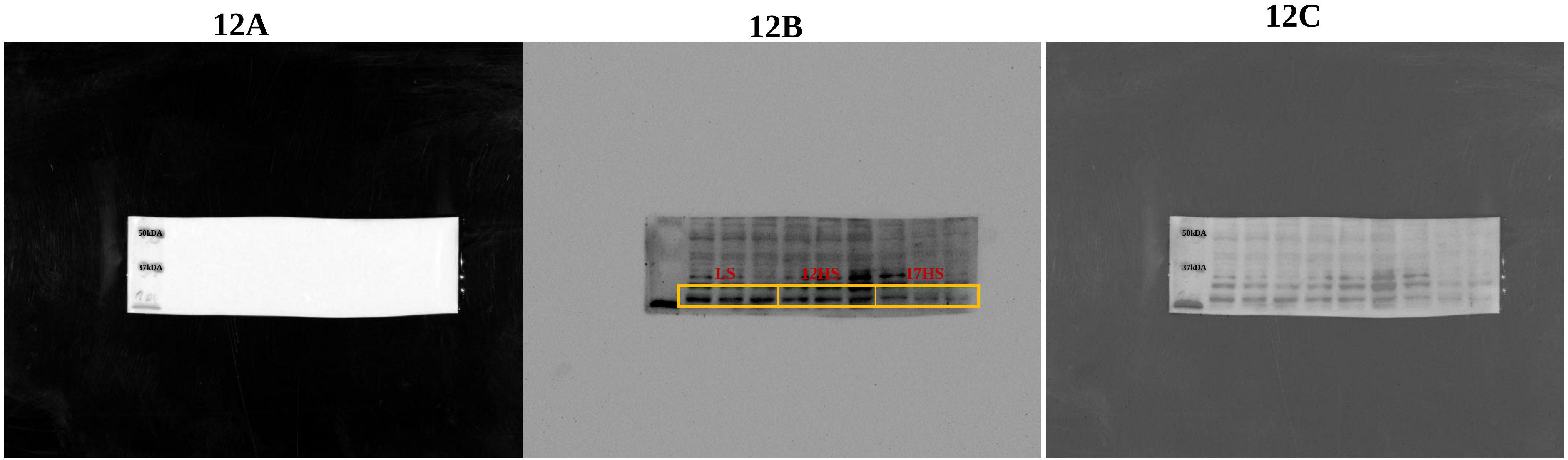

Fig.12 Original blot images of cardiac A_3R expression. *(Novus, NLS-689, 1:1000).* (Figure 12A) Colorimetric; (Figure 12B) Chemiluminescence; (Figure 12C) Merge
Yellow boxes indicate the regions used for signal quantification.

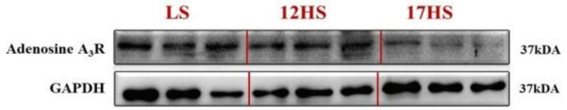

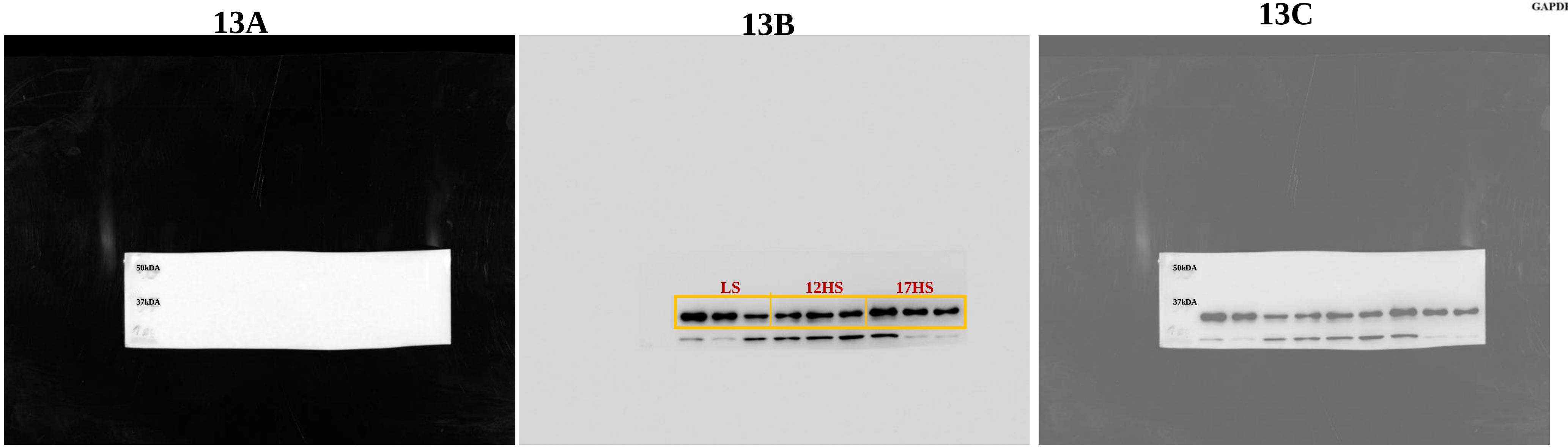

Fig.13 Original blot images of cardiac GAPDH expression. *(Sigma, G8795-100UL, 1:10000).* (Figure 13A) Colorimetric; (Figure 13B) Chemiluminescence; (Figure 13C) Merge
Yellow boxes indicate the regions used for signal quantification.

Cardiac expression of ET-1

14A

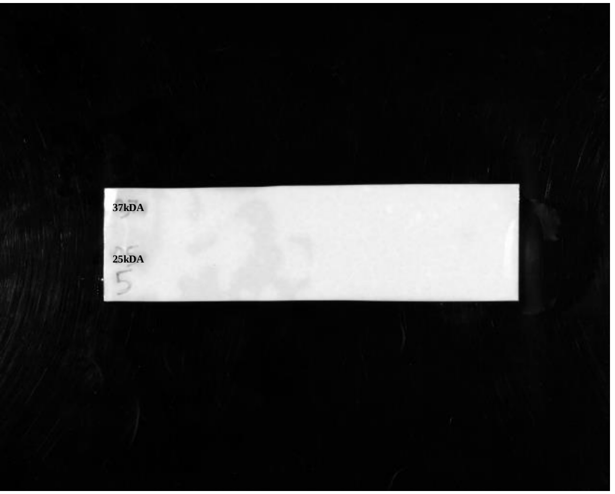

14B

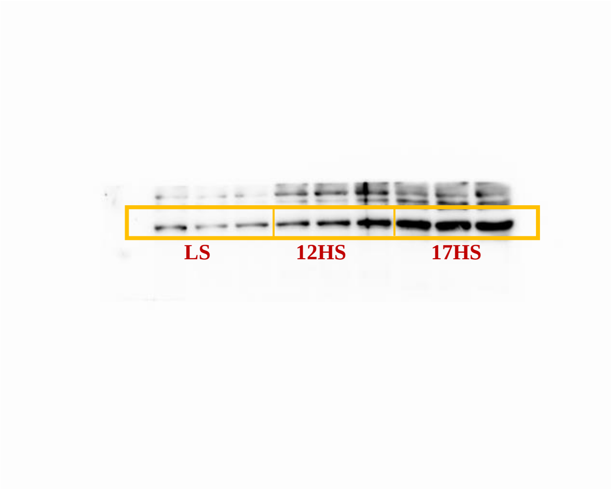

14C

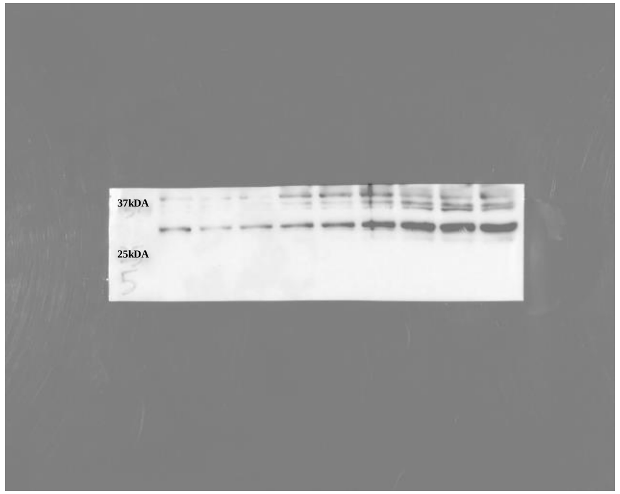

Fig.14 Original blot images of cardiac ET-1 expression. *(Abcam, ab18981, 1:1000).* (Figure 14A) Colorimetric; (Figure 14B) Chemiluminescence; (Figure 14C) Merge
Yellow boxes indicate the regions used for signal quantification.

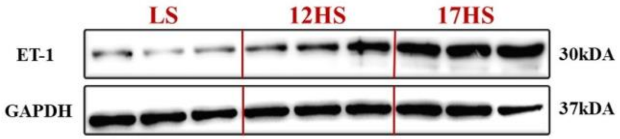

15A

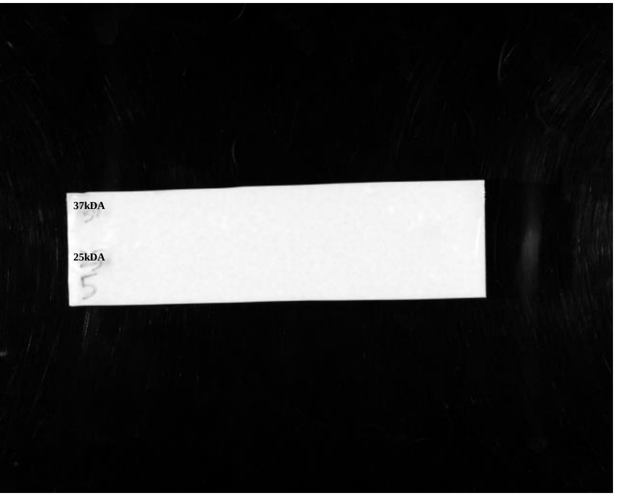

15B

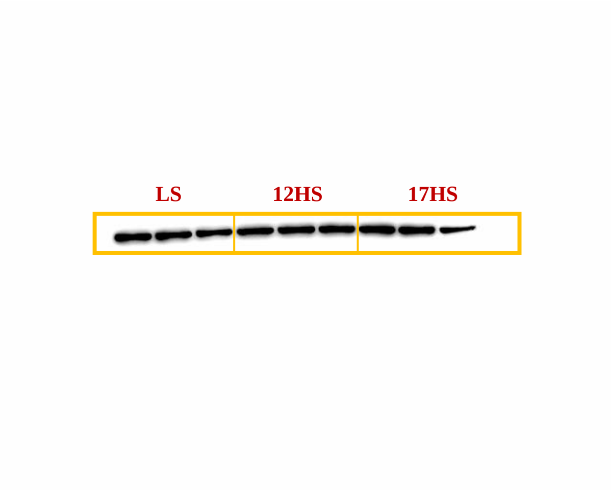

15C

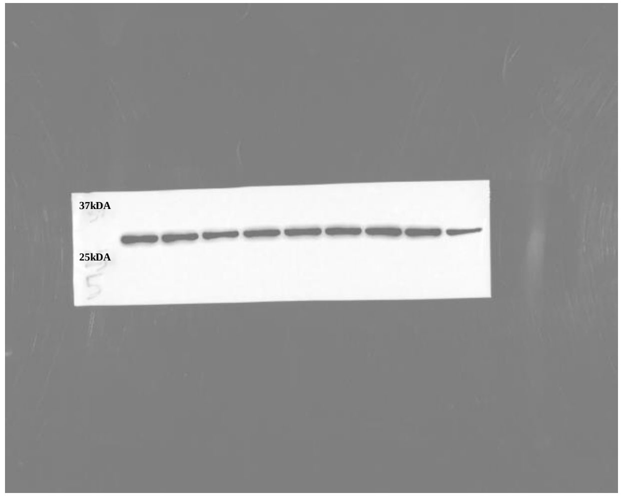

Fig.15 Original blot images of cardiac GAPDH expression. *(Sigma, G8795-100UL, 1:10000).* (Figure 15A) Colorimetric; (Figure 15B) Chemiluminescence; (Figure 15C) Merge
Yellow boxes indicate the regions used for signal quantification.

Cardiac expression of VCAM-1

16A

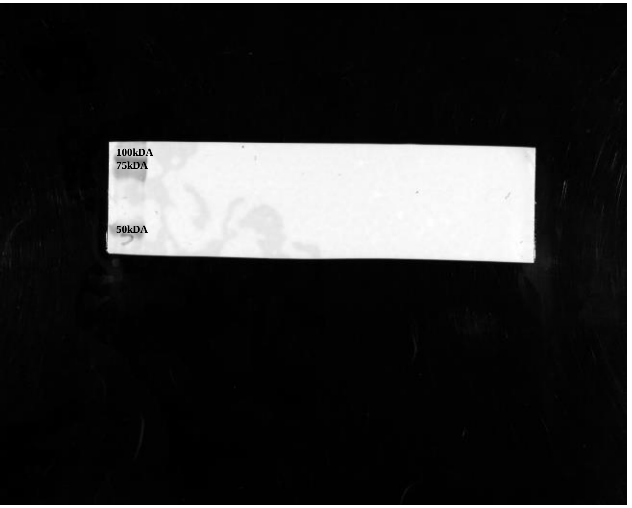

16B

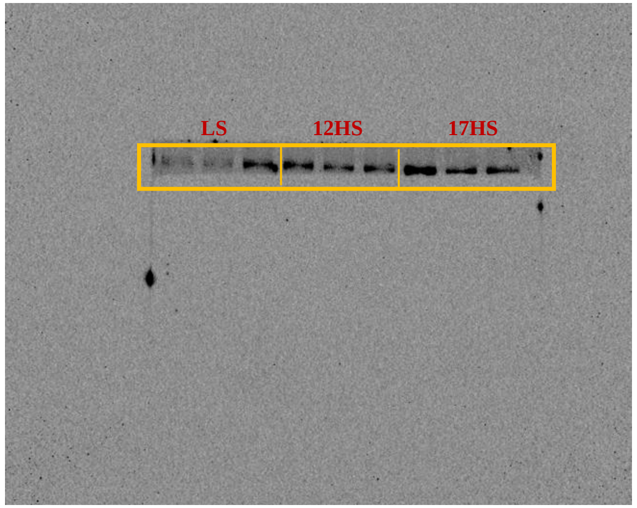

16C

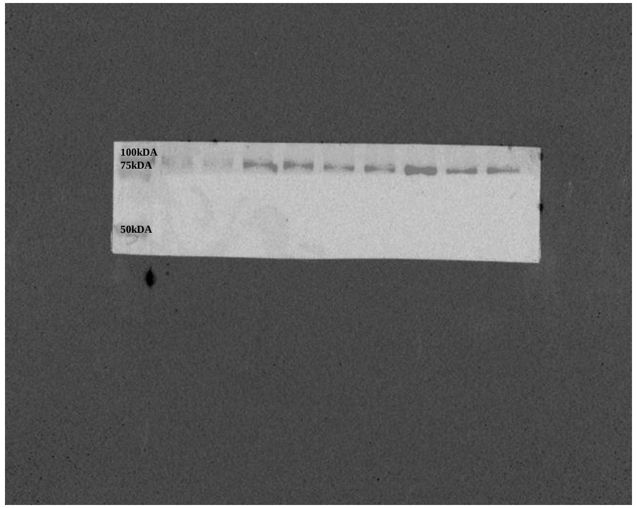

Fig.16 Original blot images of cardiac VCAM-1 expression. *(Abcam, ab134047,1:1000).* (Figure 16A) Colorimetric; (Figure 16B) Chemiluminescence; (Figure 16C) Merge Yellow boxes indicate the regions used for signal quantification.

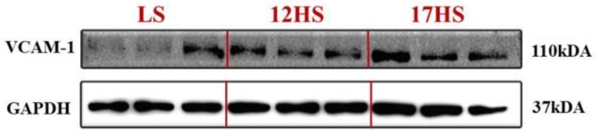

17A

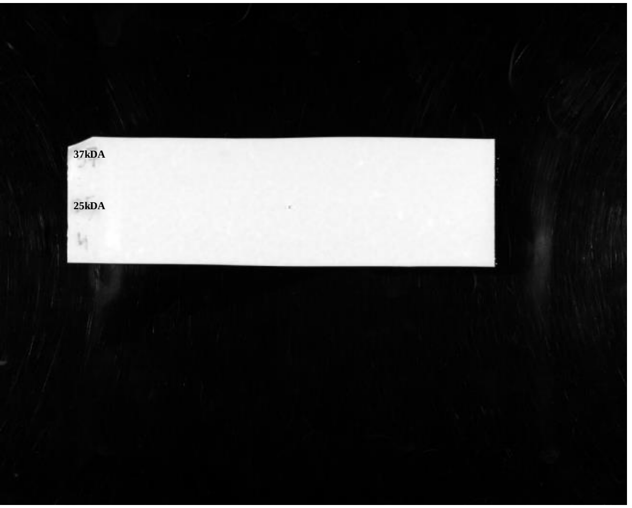

17B

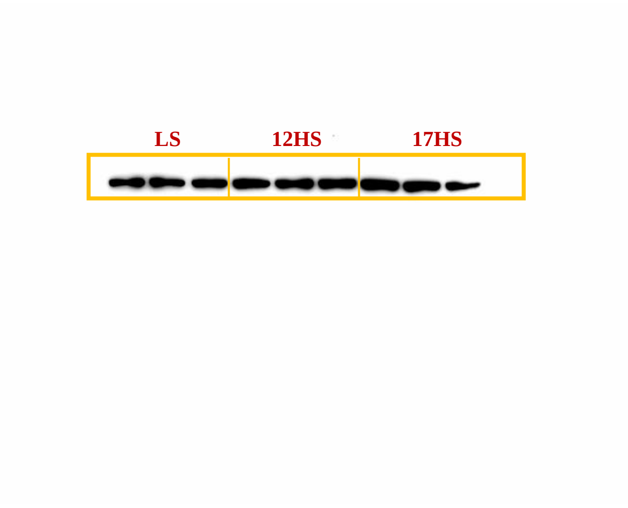

17C

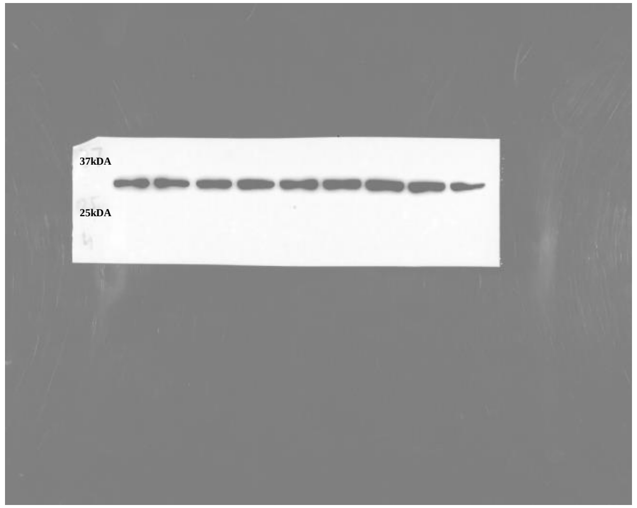

Fig.17 Original blot images of cardiac GAPDH expression. *(Sigma, G8795-100UL, 1:10000).* (Figure 17A) Colorimetric; (Figure 17B) Chemiluminescence; (Figure 17C) Merge Yellow boxes indicate the regions used for signal quantification.

Cardiac expression of ENT-2

18A

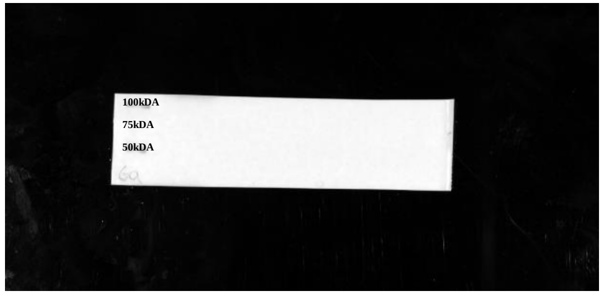

18B

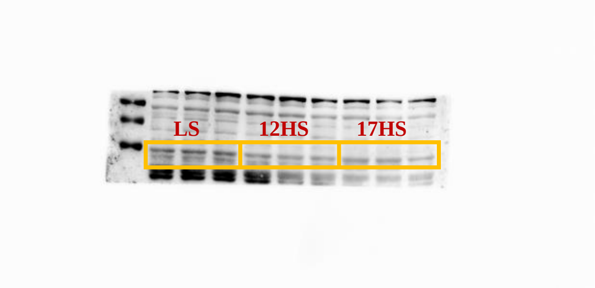

18C

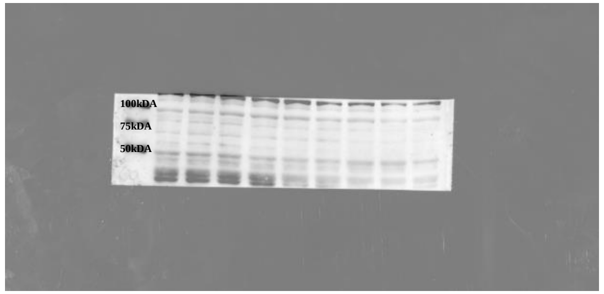

Fig.18 Original blot images of cardiac ENT-2 expression. *(Proteintech, 26082-AP, 1:500).* (Figure 18A) Colorimetric; (Figure 18B) Chemiluminescence; (Figure 18C) Merge; (Figure 18D) Full membrane. Yellow boxes indicate the regions used for signal quantification.

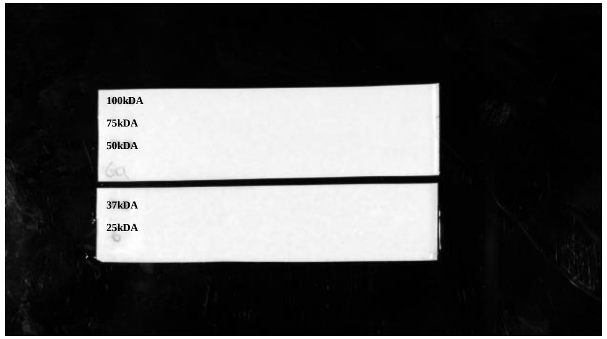

18D

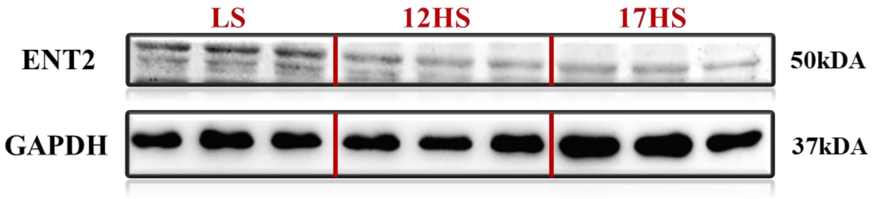

19A

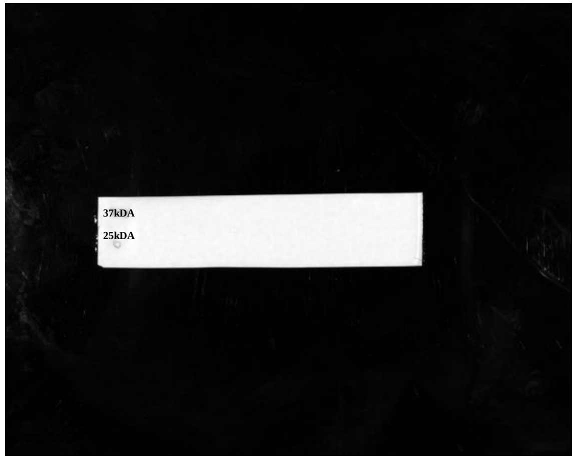

19B

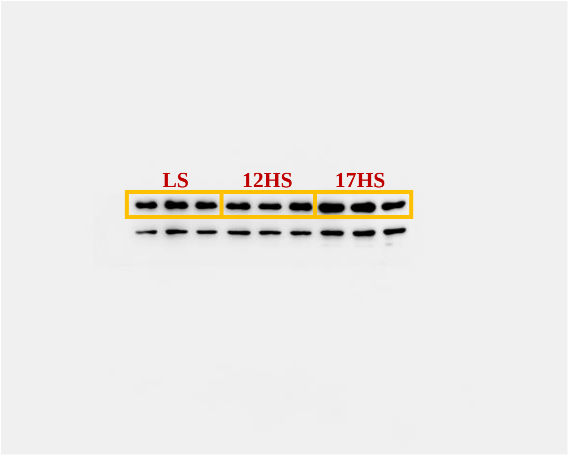

19C

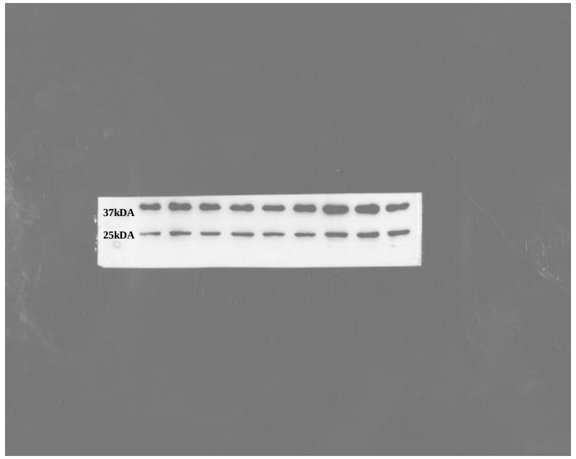

Fig.19 Original blot images of cardiac GAPDH expression. *(Sigma, G8795-100UL, 1:10000).* (Figure 19A) Colorimetric; (Figure 19B) Chemiluminescence; (Figure 19C) Merge. Yellow boxes indicate the regions used for signal quantification.

Cardiac expression of CNT2

20A

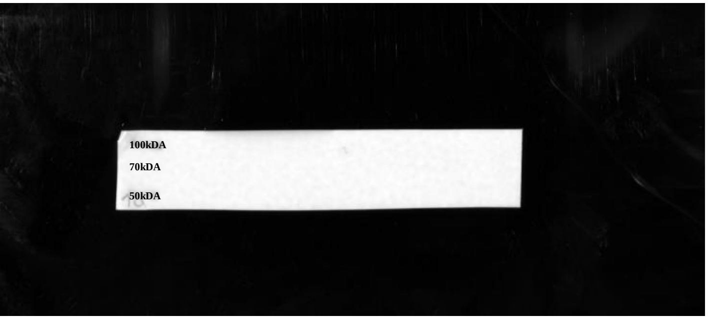

20B

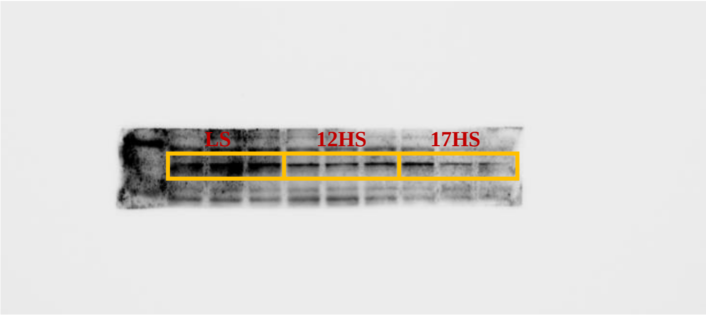

20C

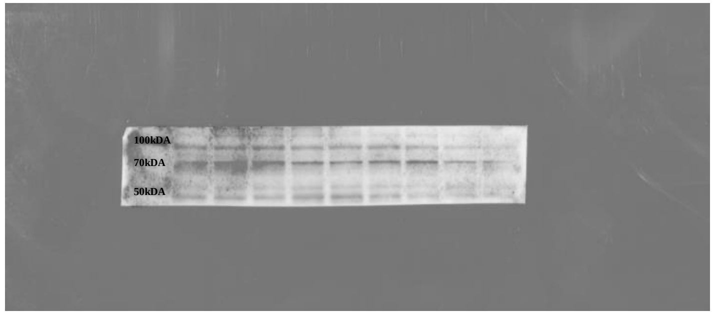

Fig.20 Original blot images of cardiac CNT2 expression. *(Invitrogen, PA5-101897, 1:500).* (Figure 20A) Colorimetric; (Figure 20B) Chemiluminescence; (Figure 20C) Merge; (Figure 20D) Full membrane. Yellow boxes indicate the regions used for signal quantification.

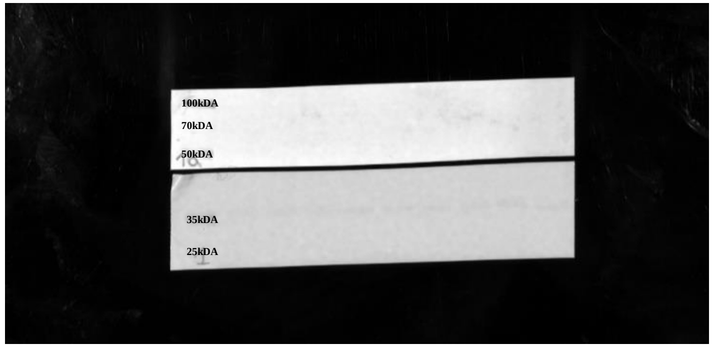

20D

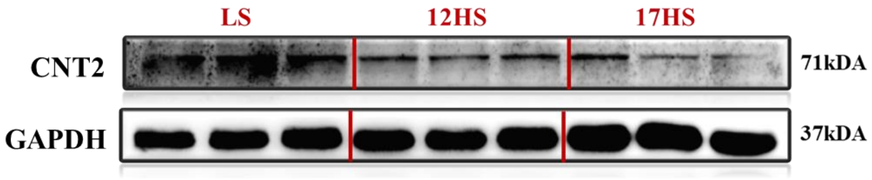

21A

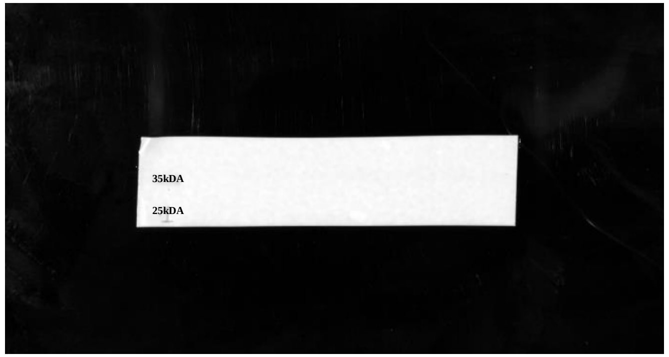

21B

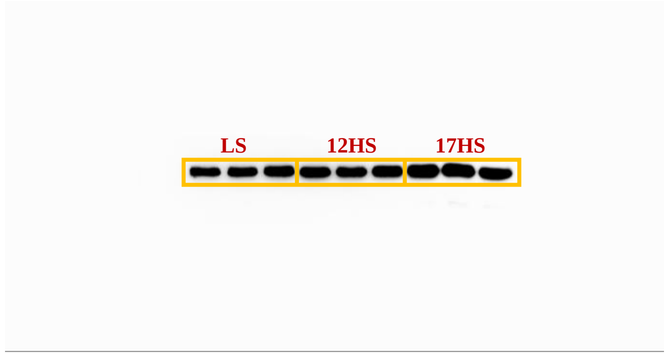

21C

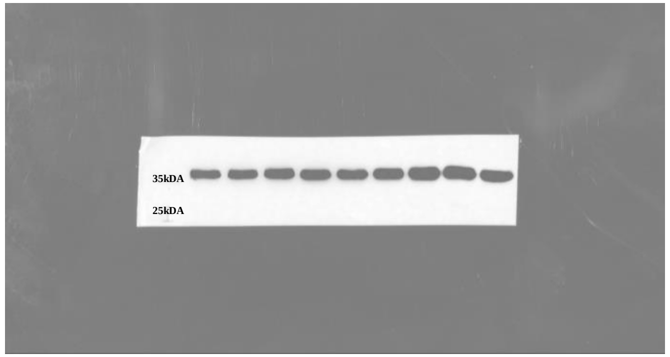

Fig.21 Original blot images of cardiac GAPDH expression. *(Sigma, G8795-100UL, 1:10000).* (Figure 21A) Colorimetric; (Figure 21B) Chemiluminescence; (Figure 21C) Merge. Yellow boxes indicate the regions used for signal quantification.

Renal expression of A_1R and $A2_AR$ cortex

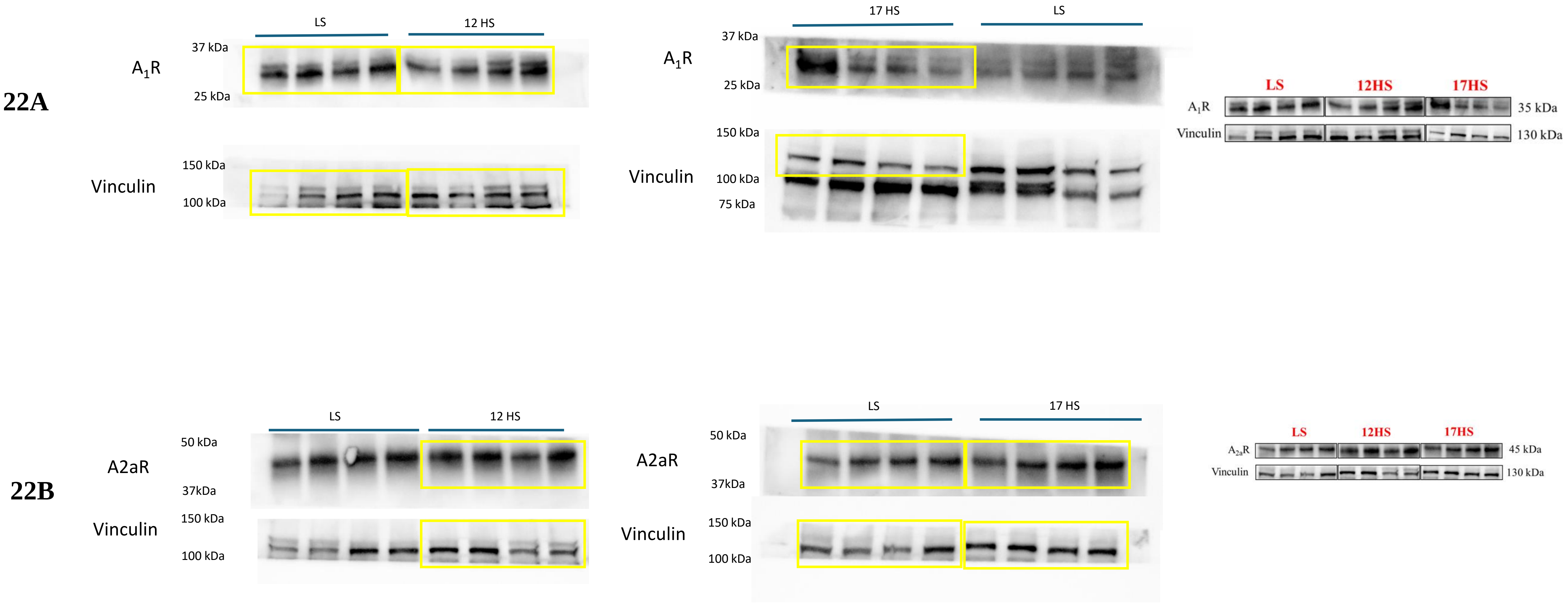

Fig. 22A and 22B Original blot images of A_1R and $A_{2A}R$ expression in renal cortex region. Primary antibodies: anti-A_1R (Santa Cruz Biotechnology, AB_2133860; 1:1000), anti-$A_{2A}R$ (Santa Cruz Biotechnology, AB_10858872; 1:1000) and anti-vinculin (Santa Cruz Biotechnology, AB_1131294; 1:1000). Yellow boxes indicate the regions used for signal quantification.

Renal expression of A_1R and $A2_AR$ outer medulla

22C

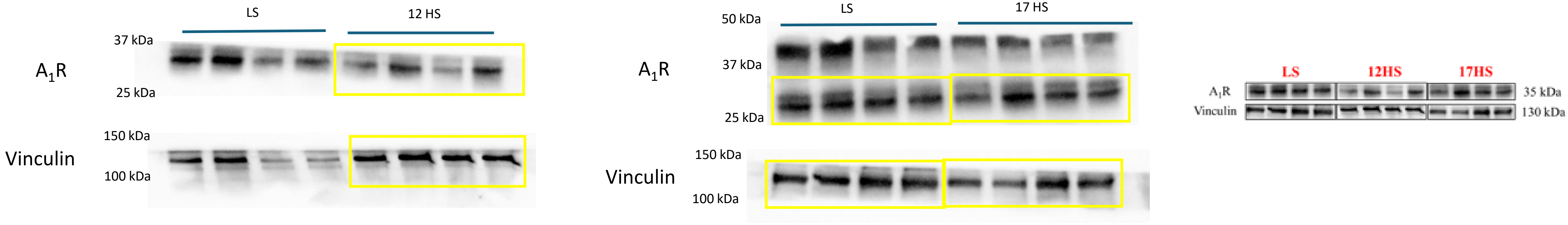

22D

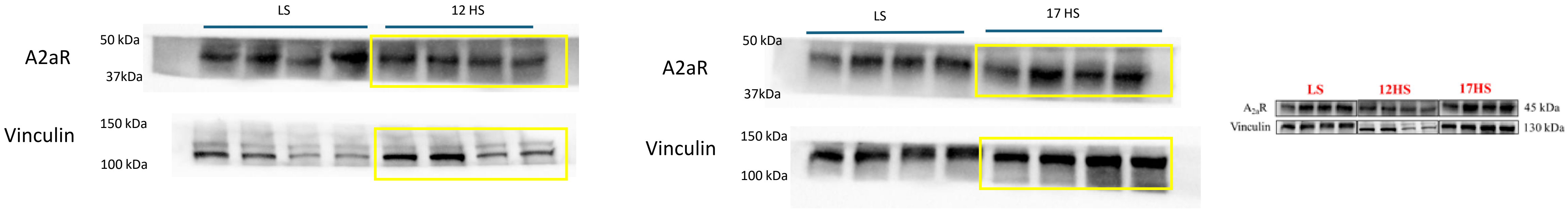

Fig. 22C and 22D Original blot images of A_1R and $A_{2A}R$ expression in the renal outer medulla fraction. Primary antibodies: anti-A_1R (Santa Cruz Biotechnology, AB_2133860; 1:1000), anti-$A_{2A}R$ (Santa Cruz Biotechnology, AB_10858872; 1:1000) and anti-vinculin (Santa Cruz Biotechnology, AB_1131294; 1:1000). Yellow boxes indicate the regions used for signal quantification.

Renal expression of A_1R and $A2_AR$ inner medulla

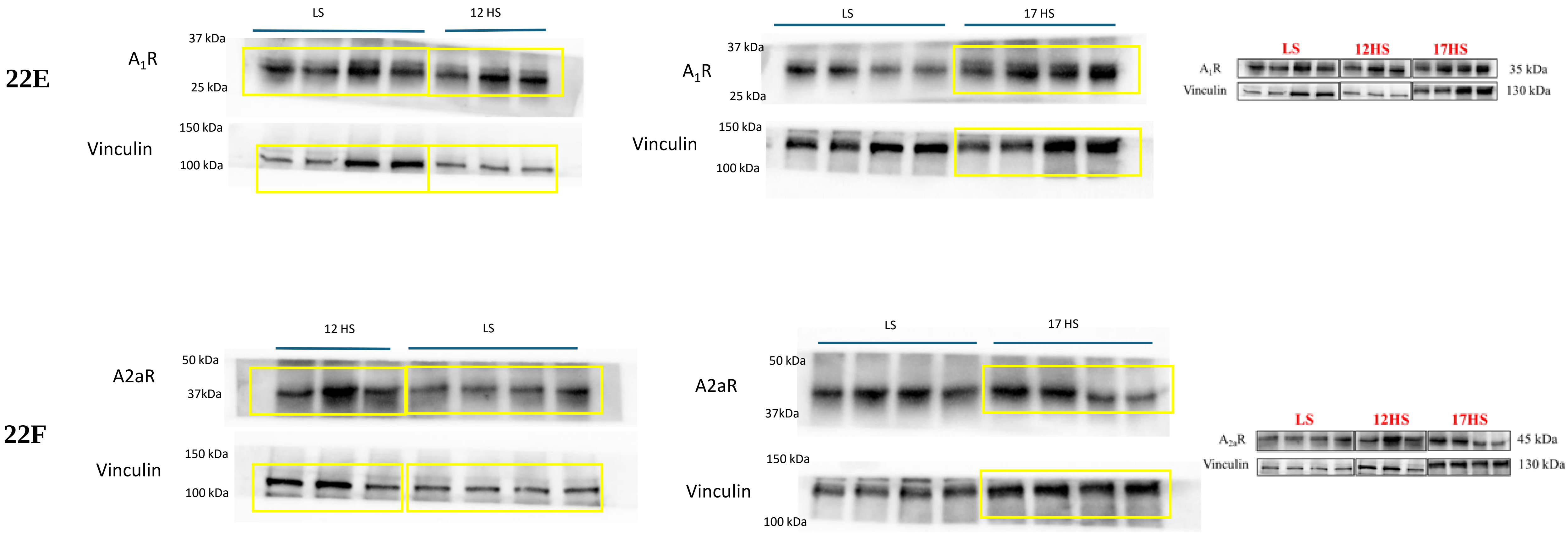

Fig. 22E and 22F Original blot images of A_1R and $A_{2A}R$ expression in the renal inner medulla fraction. Primary antibodies: anti-A_1R (Santa Cruz Biotechnology, AB_2133860; 1:1000), anti-$A_{2A}R$ (Santa Cruz Biotechnology, AB_10858872; 1:1000) and anti-vinculin (Santa Cruz Biotechnology, AB_1131294; 1:1000). Yellow boxes indicate the regions used for signal quantification.
